# Identification of Candidate mRNA and miRNA Molecules Associated with Tuberculosis Through Preliminary Analysis and Validation Using Clinical Samples

**DOI:** 10.3390/ijms27125177

**Published:** 2026-06-07

**Authors:** Yanxi Ma, Yujuan Fu, Jiahui Li, Guangyu Xu

**Affiliations:** College of Pharmacy, Beihua University, Jilin 132013, China; ma12ftbz@163.com (Y.M.); fuyujuan99@163.com (Y.F.); 15540486603@163.com (J.L.)

**Keywords:** tuberculosis, candidate molecules, microRNAs, transcriptome sequencing, RT-qPCR validation

## Abstract

Tuberculosis (TB) remains a major global public health burden. This study aimed to identify differentially expressed messenger RNAs (mRNAs) and circulating microRNAs (miRNAs) associated with TB and to validate their potential roles in the disease. We performed RNA sequencing (RNA-Seq) on peripheral blood samples from 10 patients with active pulmonary TB and 10 healthy controls, using peripheral blood mononuclear cells (PBMCs) for mRNA sequencing and plasma for miRNA sequencing. Given the exploratory nature of the plasma miRNA data and the limitations of the *U6* normalization method, the results for circulating miRNAs will need to be validated using alternative methods in subsequent experiments. A total of 1323 differentially expressed mRNAs and 49 differentially expressed miRNAs were identified. Functional annotation of differentially expressed genes was conducted using the Database for Annotation, Visualization and Integrated Discovery (DAVID), followed by Kyoto Encyclopedia of Genes and Genomes (KEGG) pathway enrichment analysis, which revealed two TB-associated pathways: “MicroRNAs in cancer” and “Small cell lung cancer.” Two key mRNAs—tumor protein p53 (*TP53*) and forkhead box protein P1 (*FOXP1*)—and one key miRNA (*hsa-miR-29b-3p*) were identified as potential core regulatory factors. Reverse transcription-quantitative polymerase chain reaction (RT-qPCR) validation confirmed that the expression patterns of these candidate molecules were consistent with the RNA-Seq results. Three potential candidate molecules associated with TB were ultimately identified, although their disease specificity remains to be determined.

## 1. Introduction

Tuberculosis (TB) is a chronic infectious disease caused by *Mycobacterium tuberculosis* (MTB), seriously endangering human health [1]. The outcome of innate host defense against MTB is determined by a multifaceted and sophisticated molecular network [2]. However, there has been a lack of systematic research on the post-transcriptional mechanisms that regulate this process [3]. This study aims to delineate the molecular regulatory networks involved in tuberculosis, thereby providing new insights into its pathogenesis and facilitating the identification of candidate molecules. We summarized the basic demographic and clinical characteristics of the study subjects (Table 1). The detailed demographic and clinical information of each participant is provided in Supplementary Table S1. Analysis revealed no statistically significant differences between the tuberculosis group and the healthy control group in terms of age (*p* = 0.53) or gender distribution (*p* = 0.07), indicating that the two groups were well-matched. All tuberculosis patients had active pulmonary tuberculosis, while none of the control subjects had a history of tuberculosis.

MicroRNAs (miRNAs) are a class of small non-coding RNAs that regulate gene expression at the post-transcriptional level [4]. By modulating the expression of target genes, they influence a range of critical cellular processes, including differentiation [5], proliferation [6], immune response [7], and apoptosis [8]. Through these regulatory functions, miRNAs play an important role in the maintenance and modulation of immune system homeostasis [9]. Given their remarkable stability in the blood [10] and correlation with disease severity [11], miRNAs have been considered as potential non-invasive biomarkers for disease diagnosis and prognosis [12].

To investigate the regulatory role of miRNAs in the pathogenesis of tuberculosis, this study employed RNA sequencing (RNA-Seq) technology to analyze differentially expressed messenger RNAs (mRNAs) and miRNAs in peripheral blood samples (peripheral blood mononuclear cells [PBMCs] for mRNA sequencing and plasma for miRNA sequencing) from patients and healthy controls. Candidate targets *TP53*/*hsa-miR-29b-3p* were validated by reverse transcription-quantitative polymerase chain reaction (RT-qPCR), whereas *LAMA2*/*hsa-miR-150-5p* did not reach statistical significance. Notably, these regulatory axes were significantly enriched in cancer-related pathways, suggesting molecular interactions between tuberculosis and oncogenic signaling pathways; this finding may offer potential insights for future diagnostic strategies.

## 2. Results

### 2.1. Differentially Expressed mRNA and miRNA

**Figure 1:**
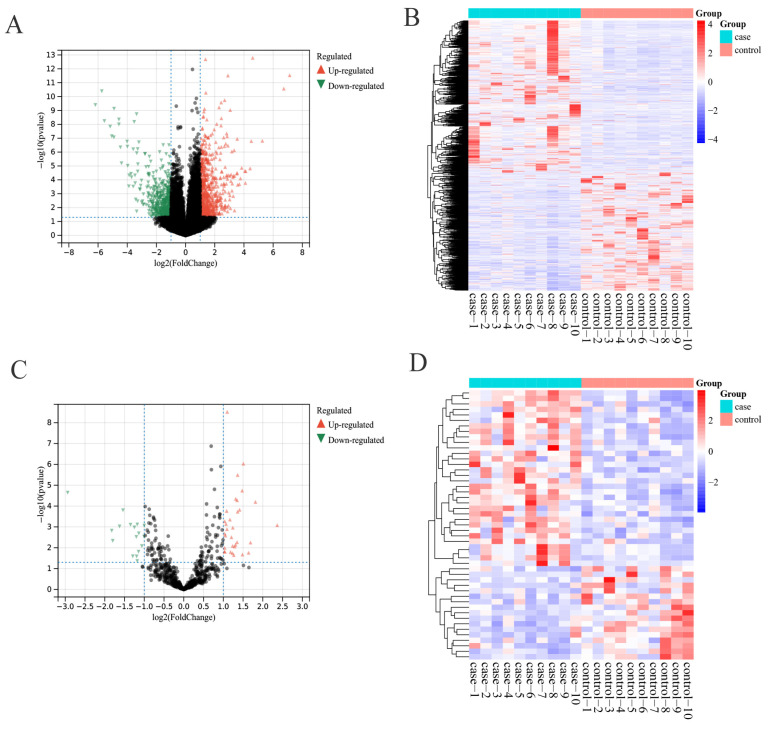
Volcano plot and heatmap of differentially expressed messenger RNAs (mRNAs) and microRNAs (miRNAs) (**A**); Heatmap of differentially expressed mRNA (**B**); Volcano plot of differentially expressed miRNA volcano map (**C**); Heatmap of differentially expressed miRNA. Case represents the tuberculosis group, and control represents the healthy control group (**D**).

The quality of the sequencing data was first evaluated. All mRNA sequencing samples yielded high raw read Phred quality scores (Q30) (both Read 1 [R1] and Read 2 [R2] > 94%) and high clean read ratios (>99%). The mapping rates to the human reference genome (Genome Reference Consortium Human Build 38 [GRCh38]) exceeded 98% for all samples (Tables S2–S4). All miRNA sequencing samples showed clean read ratios > 95% and Q30 > 95% (Table S5). These results confirm the high quality of the sequencing data, making them suitable for downstream differential expression analysis. With *p* value ≤ 0.05 and |log2 (fold change)| ≥ 1 as screening criteria, the differentially expressed mRNAs and miRNAs were screened. A total of 1323 differentially expressed mRNAs (734 upregulated and 589 downregulated) and 49 differentially expressed miRNAs (32 upregulated and 17 downregulated) were screened out (Figure 1).

**Figure 2:**
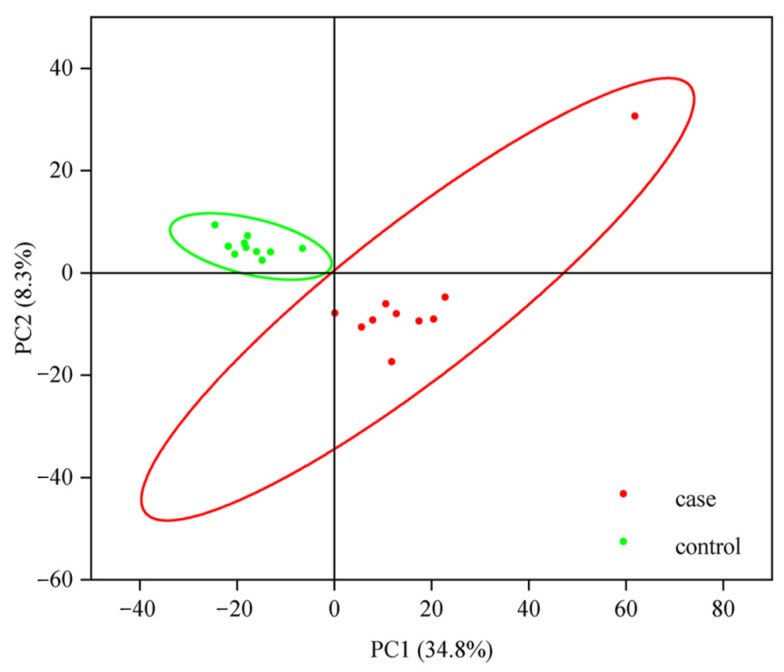
Principal Component Analysis (PCA). Green represents the healthy control group, and red represents the tuberculosis group.

Principal Component Analysis (PCA) is the most widely used data dimensionality reduction algorithm, and it measures the variability of data using variance and projects high-dimensional data with significant variability into a low-dimensional space for representation. Our PCA results showed that the blood samples of tuberculosis patients were significantly separated from the blood samples of healthy controls (PC1 was 34.8%, and PC2 was 8.3%) (Figure 2).

### 2.2. Screened Key mRNAs

**Figure 3:**
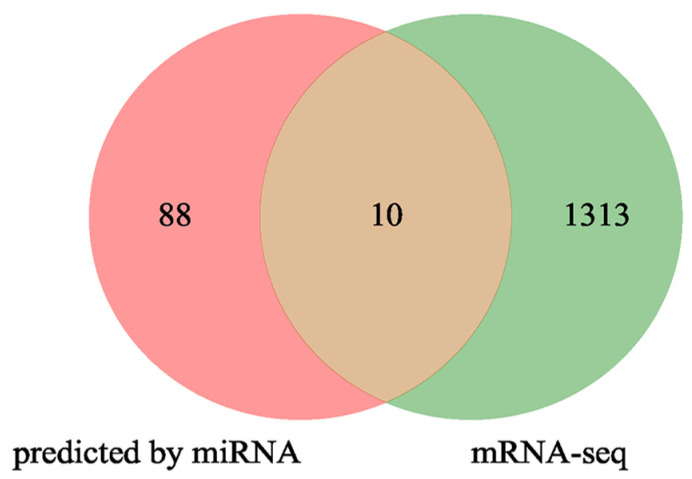
Venn diagram of the intersection between mRNAs for miRNA prediction and mRNAs by messenger RNA sequencing (mRNA-Seq). The red color refers to mRNAs by miRNA prediction, and the green color shows mRNAs by mRNA-Seq.

The miRWalk database was used to predict the target genes of 49 differentially expressed miRNAs screened by RNA-seq, and a total of 98 corresponding target genes were obtained after removing duplicates (Table S6). The predicted 98 target genes were intersected with 1323 differentially expressed mRNAs screened by RNA-seq, and 10 key mRNAs (*EIF5*, *TP53*, *TSPAN3*, *CSGALNACT1*, *AAK1*, *DNMT3A*, *LAMA2*, *MXD1*, *TNFAIP8* and *FOXP1*) were identified (Figure 3, Table 2).

### 2.3. KEGG Analysis of Key mRNAs

**Figure 4:**
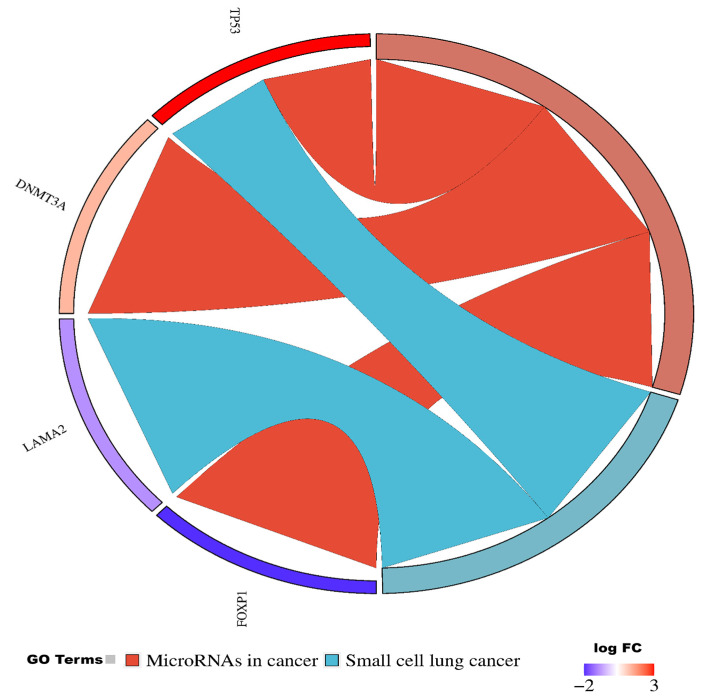
KEGG Pathway Enrichment Analysis of Key mRNAs.

Kyoto Encyclopedia of Genes and Genomes (KEGG) pathway enrichment analysis on the 10 selected key mRNAs was conducted (Figure 4). The results showed that these 10 key mRNAs were only present in two pathways (microRNAs in cancer and small cell lung cancer). It should be noted that these two pathways are not specific to tuberculosis. This enrichment result may be due to the small sample size or to nonspecific responses shared between tuberculosis and cancer. This study did not include other pulmonary diseases (such as lung cancer, pneumonia, or chronic obstructive pulmonary disease [COPD]) as controls; therefore, it is not possible to determine whether these pathway associations are specific to tuberculosis. In summary, the results of this enrichment analysis should be considered preliminary observations and require further validation in larger cohorts that include a variety of pulmonary diseases.

### 2.4. RT-qPCR Verification

**Figure 5:**
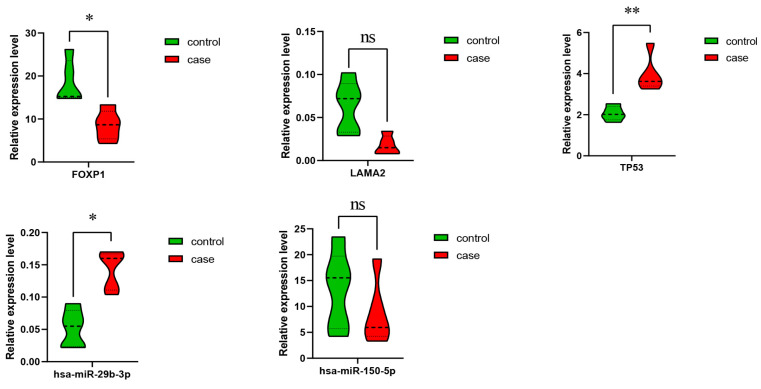
RT-qPCR analysis of mRNA and miRNA data. *, *p* ≤ 0.05; **, *p* ≤ 0.01; ns, *p* > 0.05.

RT-qPCR validation was performed on three mRNAs (*TP53*, *LAMA2*, and *FOXP1*) and two miRNAs (*hsa-miR-29b-3p* and *hsa-miR-150-5p*). The RT-qPCR results were not completely consistent with the sequencing results: *FOXP1* was significantly downregulated, and *TP53* and *hsa-miR-29b-3p* were significantly upregulated, whereas no significant changes were observed for *LAMA2* and *hsa-miR-150-5p* (Figure 5).

## 3. Discussion

The early diagnosis and treatment of tuberculosis is still a huge challenge to global public health [13], and finding new tuberculosis candidate molecules is the key to the early diagnosis and following treatment of tuberculosis. In this study, we systematically screened for potential molecular targets associated with tuberculosis using whole-transcriptome sequencing (RNA-Seq). This integrated analysis provides a novel theoretical and experimental foundation for understanding TB pathogenesis and for developing early diagnostic strategies.

In this study, 1323 differentially expressed mRNAs and 49 differentially expressed miRNAs were identified by RNA-Seq screening first. Subsequently, mRNA target genes were predicted for differentially expressed miRNAs and intersected with mRNAs screened by RNA-seq to find 10 key mRNAs.

There are three mRNAs (*FOXP1*, *TP53*, and *LAMA2*), among which *LAMA2* expression is not significant. *FOXP1* is a member of the FOXP subfamily, and *FOXP1* can regulate the activity of transcription factors [14], activate T cells [15], and enhance autoimmune function [16]. The *TP53* signaling pathway is involved in regulating the occurrence of many common cancers in humans [17]. *TP53* can activate the tumor necrosis factor-α (TNF-α) [18] to coordinate with the Sonic Hedgehog (SHH) signaling pathway to regulate the homeostasis of cells [19]. It has been found that the *TP53* signaling pathway is also involved in the immune regulation process of pathogenic bacteria (such as MTB) infection in the body [20]. Gene *LAMA2* has also been reported to be associated with the immune system [21]. The early characteristic of *LAMA2* deficiency is the increased expression of pro-inflammatory cytokine TNF-α and interleukin-1 beta (IL-1β) [22]. The production of inflammatory factors is believed to be an immune evasion mechanism in the interaction of MTB with the host [23]. To sum up, these three mRNAs may be closely related to the host immune system. Furthermore, the downregulation of the *FOXP1* gene suggests that its expression may be suppressed in tuberculosis patients, leading to weakened immunity and subsequently triggering latent *Mycobacterium tuberculosis* infection.

MiRNAs can play an important regulatory role in both acquired and innate immunity [24], with a fine regulatory effect on the body’s autoimmune system, preventing an excessive immune activation of the body [25]. We identified two miRNAs (*hsa-miR-29b-3p* and *hsa-miR-150-5p*), of which *hsa-miR-150-5p* was not significantly expressed. *Hsa-miR-29b-3p* has been confirmed to be differentially expressed in lung cancer, typically downregulated in an advanced lung cancer and upregulated in an early lung cancer, so it can be used as a diagnostic biomarker for lung cancer [26]. The abnormal expression of *miR-150* can cause some immune system diseases [27], and *hsa-miR-150-5p* is involved in various cellular functions in different types of cells and has also been found to regulate cell cycle progression, proliferation, tumor occurrence, and so on [28]. MTB infection and cancers are two diseases that tend to produce resistance to the host immune system. Some studies have shown that tuberculosis and cancers have certain similarities in immune regulation [29]. Since *hsa-miR-29b-3p* is associated with both immunity and cancer, and our validation results are consistent with sequencing data, this miRNA may represent a potential candidate molecule for tuberculosis.

The 10 key mRNAs were only enriched in two pathways (microRNAs in cancer and small cell lung cancer pathways). The molecular mechanisms underlying changes in the small cell lung cancer pathway include the expression of protooncogene and the deficiency of tumor suppressor genes, and microRNAs in cancer pathway also shows that many miRNAs are involved in the infection process of tuberculosis, indicating that these two pathways are also involved in the regulation of cancer and tuberculosis infection. A systematic review and meta-analysis indicated that individuals with a history of tuberculosis have a significantly increased risk of lung cancer (adjusted risk ratio 1.51, 95% confidence interval 1.30–1.76), particularly within the first two years following a tuberculosis diagnosis (risk ratio 5.01) [30]. The authors attributed this association to chronic inflammation caused by *Mycobacterium tuberculosis* infection, which can generate reactive oxygen/nitrogen species, lead to deoxyribonucleic acid (DNA) damage, and activate oncogenic signaling pathways. Therefore, although our KEGG enrichment analysis identified only cancer-related pathways (microRNAs in cancer and small cell lung cancer), this observation is consistent with the concept that tuberculosis-associated chronic inflammation may trigger nonspecific carcinogenic signals.

We also observed that the expression levels of *hsa-miR-29b-3p* and *LAMA2* were negatively correlated, and that the expression levels of *hsa-miR-150-5p* and *TP53* were also negatively correlated. Furthermore, *TP53* and *LAMA2* are both present in the small cell lung cancer pathway. Meanwhile, both *TP53* and *FOXP1* existed in the microRNAs in the cancer pathway. Therefore, it is speculated that the three genes may be involved in the regulation of microRNAs in cancer and small cell lung cancer pathways, ultimately leading to the occurrence of tuberculosis. However, the expression results for *TP53*, *LAMA2*, *FOXP1*, *hsa-miR-29b-3p*, and *hsa-miR-150-5p* are not fully consistent with the RNA-Seq findings; specifically, the expression levels of *LAMA2* and *hsa-miR-150-5p* were not significant, which may be attributed to the small sample size. In addition, *TP53*, *LAMA2* and *FOXP1* are all associated with mitochondrial dysfunction. *Hsa-miR-150-5p* can increase the expression of *TP53*, and mutated *TP53* can regulate the metabolism and function of mitochondria [31]. *Hsa-miR-29b-3p* can inhibit the expression of *LAMA2* and is expressed by the extracellular matrix (ECM) in the small cell lung cancer pathway, while mitochondrial dysfunction will affect the composition of the ECM [32], and the related ECM–receptor interaction pathway has been proved to be related to tuberculosis [33]. Moreover, the lack of *FOXP1* has also been proven to be associated with mitochondrial dysfunction [34]. To sum up, we speculate that the occurrence of tuberculosis may be related to both the immune system and energy metabolism.

In this study, *U6* small nuclear RNA (*U6*) was used as an internal control for miRNA quantification. However, *U6* is a nuclear transcript that is unstable and present at low concentrations in cell-free samples such as plasma and serum. Therefore, it is not a suitable internal control for studies of circulating miRNAs. This may affect the accuracy of miRNA expression quantification. Consequently, the relevant miRNA results in this study should be considered preliminary observations. Future studies should use established internal controls (such as *miR-16* and *miR-93*) or incorporate exogenous controls (such as *cel-miR-39*) to ensure data reliability.

## 4. Materials and Methods

### 4.1. Human Sample Acquisition

In this study, 20 blood samples were collected, including 10 patients with pulmonary tuberculosis and 10 healthy controls. These 20 volunteers were all over 20 years old, and were excluded from the past history of malignant tumors. This study was approved by the Medical Ethics Committee of Jilin Provincial Tuberculosis Hospital. All 20 participants provided written informed consent to participate in the study. We have uploaded the RNA transcriptome sequencing data to the National Center for Biotechnology Information (NCBI) database. RNA-Seq data can be obtained at ID 876021-BioProject-NCBI (nih.gov). Available clinical characteristics of these patients are summarized (Table S7).

### 4.2. mRNA and miRNA Sequencing and Data Analysis

First, peripheral blood mononuclear cells (PBMCs) were isolated from whole blood samples by Ficoll-Paque (GE Healthcare, Chicago, IL, USA) density gradient centrifugation. Total RNA was then extracted and purified from the isolated PBMCs using TRIzol reagent (Invitrogen, Carlsbad, CA, USA). To remove residual genomic DNA (gDNA) that could interfere with downstream reverse transcription and quantitative PCR (qPCR) quantification (especially for the *U6* internal control, which contains multiple pseudogenes), we treated the RNA samples with DNase I. The procedure is as follows: Mix 1 μg of total RNA with 1 μL of gDNA Eraser (from the PrimeScript^TM^ RT Kit, RR047A; Takara Bio Inc., Kusatsu, Shiga, Japan) to a total volume of 10 μL, incubate at 42 °C for 2 min, and then immediately place on ice. This step effectively degrades gDNA. After DNase treatment, RNA quality was assessed using an Agilent Bioanalyzer 2100 (Agilent Technologies, Santa Clara, CA, USA). Only RNA samples with an RNA integrity number (RIN) ≥ 7.0 were selected to ensure the construction of high-quality total RNA sequencing libraries for downstream analysis. The sequencing criteria were as follows: RNA concentration ≥ 100 ng/µL, total RNA amount > 2 μg, OD260/280 ratio between 1.8 and 2.2, and OD260/230 ≥ 2.0. Finally, the libraries were sequenced using a 2 × 150 bp paired-end sequencing strategy. For miRNA sequencing, we prepared small RNA libraries using the TruSeq small RNA Sample Preparation Kit (Illumina, San Diego, CA, USA). The specific procedure was as follows. Total RNA (≥200 ng/µL, RIN ≥ 7) was sequentially ligated with T4 RNA ligase 2 (truncated) and T4 RNA ligase (both from New England Biolabs, Ipswich, MA, USA) to attach 3′ and 5′ adapters, respectively. Reverse transcription was performed using SuperScript^TM^ IV Reverse Transcriptase (Thermo Fisher Scientific, Waltham, MA, USA), followed by polymerase chain reaction (PCR) amplification (12–15 cycles) to enrich complementary DNA (cDNA) fragments containing the adapter sequences. The small RNA libraries were size-selected (140–160 bp) by 6% polyacrylamide gel electrophoresis (PAGE), purified, and quantified using an Agilent 2100 Bioanalyzer. Finally, the libraries were sequenced on an Illumina HiSeq 2500 platform (Illumina, San Diego, CA, USA) with 2 × 150 bp paired-end reads. To confirm the specificity of the *U6* primer (which served as an internal control for miRNA quantification), we performed an NCBI Primer-Basic Local Alignment Search Tool (BLAST) analysis (https://blast.ncbi.nlm.nih.gov/Blast.cgi accessed on 23 October 2022). The primer pair matched multiple internal *U6* pseudogenes, including the *KIF1B* gene on chromosome 1 and the *TMEM222* gene on chromosome 2. Therefore, rigorous DNase I treatment was performed prior to reverse transcription. In addition, each qPCR experiment included a no-reverse-transcription (NRT) control (i.e., using pure water in place of reverse transcriptase). No cycle threshold (Ct) values were detected in any of the NRT controls. The melting curve for *U6* exhibited a single sharp peak (Figure S1), confirming that the amplification signal originated entirely from the *U6* transcript and not from genomic DNA.

FastQC (v0.11.8) and R (v3.5.1) was used to evaluate the quality of raw sequencing data. Adapter sequences and low-quality bases (Q < 10) were trimmed from both ends using TrimGalore (v0.6.7). Reads shorter than 35 bp after trimming were discarded. These steps were applied to both R1 and R2 reads. The resulting clean reads were used for all downstream analyses.

The differentially expressed genes of volunteers in tuberculosis group (case group) and healthy control group (control group) were analyzed using Deseq2 (v1.10.1). Type was the screening result, and after adjusting the gene expression profile, genes with *p* values < 0.05 and |log2 (fold change)| ≥ 1 were considered the differentially expressed genes (Table S8).

### 4.3. Screening of mRNA and miRNA

The target gene mRNA of differentially expressed miRNAs screened by RNA-seq was predicted in miRWalk database at http://miRWalk.umm.uni-heidelberg.de/ (accessed on 23 October 2022) [35], the predicted mRNAs were intersected with the mRNAs obtained by RNA-seq.

### 4.4. KEGG Pathway Enrichment Analysis

The hinge genes were submitted to the Database for Annotation, Visualization and Integrated Discovery (DAVID) at https://davidbioinformatics.nih.gov/, and *Homo sapiens* was taken as the research object [36]. We made use of Kyoto Encyclopedia of Genes and Genomes (KEGG) to conduct the hinge gene pathway classification.

### 4.5. RT-qPCR Verification Procedures

Total RNA was extracted from PBMCs using the TRNzol method, and its concentration was measured using a NanoDrop^TM^ One spectrophotometer (Thermo Fisher Scientific, Waltham, MA, USA). In this study, the stem-loop method was used for miRNA reverse transcription. Specific reverse transcription primers (designed based on the sequences of *miR-29b-3p* and *miR-150-5p*) were used according to the manufacturer’s instructions (TransGen Biotech, Beijing, China). The RNA samples from each group were subjected to reverse transcription and detected by PCR according to the instructions of miRNA-specific reverse transcription reagent kit from Beijing TransGen Biotech Co., Ltd. (Beijing, China) and ChamQ SYBR qPCR Master Mix reagent kit from Vazyme Biotech Co., Ltd. (Nanjing, China). The PCR amplification was performed using a StepOne^TM^ Real-Time PCR System (Applied Biosystems, Foster City, CA, USA). The PCR reaction system was 20 μL, and the reaction conditions were 95 °C for 5 min, 95 °C for 15 s, 58 °C for 15 s, and 70 °C for 15 s, with a total of 40 cycles. Relative expression levels were calculated using the $2^{-\Delta \Delta Ct}$ method. First, ΔCt values were calculated as $\Delta Ct={Ct}_{target\;gene}-{Ct}_{internal\;reference\;gene}$ (*GAPDH* was used as the internal control for mRNA, and *U6* was used as the internal control for miRNA). In this study, the healthy control group served as the calibration standard, with the mean ΔCt value of the healthy control group used as the baseline. ΔΔCt was calculated as $\Delta \Delta Ct={\Delta Ct}_{samples\;from\;tuberculosis\;patients}-\bar{{\Delta Ct}_{healthy\;control\;group}}$. The fold change of each target in tuberculosis patients relative to the healthy control group is calculated as $2^{-\Delta \Delta Ct}$. A fold change > 1 is considered upregulated, and <1 is considered downregulated. (Table 3). In addition, melting curve analysis confirmed the specificity of each amplicon (single peak), as shown in Figure S1.

## 5. Conclusions

In summary, through RNA-Seq analysis, this study identified three candidate molecules (*TP53*, *FOXP1* and *hsa-miR-29b-3p*) in tuberculosis. Notably, these molecules are primarily enriched in cancer-related pathways, suggesting unexpected molecular interactions between the pathogenesis of tuberculosis and oncogenic signaling. These findings highlight potential molecular targets and provide new insights for the development of improved diagnostic tools for tuberculosis. However, due to technical limitations in *U6* normalization, plasma miRNA data are currently still in the exploratory phase; therefore, these results need to be validated using alternative methods in future experiments. Larger-scale studies and functional validation are required to confirm the potential clinical relevance of these candidate molecules.

## Figures and Tables

**Table 1:** Baseline characteristics of tuberculosis (TB) patients and healthy controls.

Characteristic	TB Patients (*n* = 10)	Healthy Controls (*n* = 10)	*p* Value
Age (years, mean ± standard deviation [SD])	49.2 ± 16.0	45.6 ± 7.3	0.5283757
Sex (male/female)	8/2	3/7	0.06977852
Pulmonary tuberculosis (active)	10	0	N/A

**Table 2:** Ten key mRNAs.

No.	Gene Symbol	log2 Fold Change	*p* Value
1	*EIF5*	1.550223837	0.001095924
2	*TP53*	2.787677009	0.000192395
3	*TSPAN3*	−1.108075776	0.006530098
4	*CSGALNACT1*	1.914176985	0.019506938
5	*AAK1*	2.494081353	0.026885414
6	*DNMT3A*	1.043820128	0.043709217
7	*LAMA2*	−1.330648879	1.39024 × 10^−6^
8	*MXD1*	1.325623476	0.000217589
9	*TNFAIP8*	−1.085659727	0.003859843
10	*FOXP1*	−2.482678899	2.13111 × 10^−6^

**Table 3:** Primer sequences.

Name	Forward Primer	Reverse Primer
*FOXP1*	AACCTCTTGCTCAAGGCATGATT	GCTGTGATTGTTGCCTGTGG
*TP53*	GCGCTTCGAGATGTTCCGAG	ATGGCGGGAGGTAGACTGAC
*LAMA2*	ACCCGAAGAATTGGTCCAGTGA	GGTTGTTCCAGATCGGCAGG
*hsa-miR-150*	GCGGTCTCCCAACCCTTGTA	GTCGTATCCAGTGCAGGGTCCGAGGTATTCGCACTGGATACGACCACTGG
*hsa-miR-29b-3p*	GGCGCTAGCACCATTTGAAATC	GTCGTATCCAGTGCAGGGTCCGAGGTATTCGCACTGGATACGACAACACT
*GAPDH*	GGAGTCCACTGGCGTCTTCA	GCAGAGGGGGCAGAGATGAT
*U6*	GTGCTCGCTTCGGCAGCACATA	GCGCAGGGGCCATGCTAATCTTC

## Data Availability

The data that support the findings of this study are openly available in the NCBI BioProject database at https://www.ncbi.nlm.nih.gov/bioproject/PRJNA876021, reference number PRJNA876021, accessed on 5 June 2026.

## Supplementary Materials

The following supporting information can be downloaded at: https://www.mdpi.com/article/10.3390/ijms27125177/s1.

## Abbreviations

The following abbreviations are used in this manuscript:

TB	Tuberculosis
MTB	*Mycobacterium tuberculosis*
miRNA	microRNA
mRNA	messenger RNA
RNA-Seq	RNA sequencing
RT-qPCR	reverse transcription-quantitative polymerase chain reaction
KEGG	Kyoto Encyclopedia of Genes and Genomes
DAVID	Database for Annotation, Visualization and Integrated Discovery
PCA	Principal Component Analysis
PBMCs	Peripheral Blood Mononuclear Cells
COPD	Chronic Obstructive Pulmonary Disease
NCBI	National Center for Biotechnology Information
BLAST	Basic Local Alignment Search Tool
PCR	Polymerase Chain Reaction
cDNA	Complementary DNA
Ct	Cycle Threshold
R1	Read 1
R2	Read 2
Q30	Phred Quality Score 30
GRCh38	Genome Reference Consortium Human Build 38
TNF-α	Tumor Necrosis Factor-alpha
SHH	Sonic Hedgehog
ECM	Extracellular Matrix
IL-1β	Interleukin-1 beta
