# Supplementary material for: Identification of Candidate mRNA and miRNA Molecules Associated with Tuberculosis Through Preliminary Analysis and Validation Using Clinical Samples
Source: Int J Mol Sci. 2026 Jun 7;27(12):5177. doi: 10.3390/ijms27125177 (PMC13299930; doi:10.3390/ijms27125177)
Supplement: Supplementary file 1 [file ijms-27-05177-s001.zip › Table S1.pdf]

Table S1. Detailed demographic and clinical information of individual participants.

Subject ID	Age (years)	Sex	Disease status
TB-1	36	Male	Active pulmonary tuberculosis
TB-2	71	Male	Active pulmonary tuberculosis
TB-3	47	Female	Active pulmonary tuberculosis
TB-4	22	Male	Active pulmonary tuberculosis
TB-5	67	Male	Active pulmonary tuberculosis
TB-6	59	Male	Active pulmonary tuberculosis
TB-7	35	Female	Active pulmonary tuberculosis
TB-8	39	Male	Active pulmonary tuberculosis
TB-9	62	Male	Active pulmonary tuberculosis
TB-10	54	Male	Active pulmonary tuberculosis
HC-1	32	Male	Healthy
HC-2	46	Female	Healthy
HC-3	37	Female	Healthy
HC-4	54	Male	Healthy
HC-5	50	Female	Healthy
HC-6	54	Female	Healthy
HC-7	40	Female	Healthy
HC-8	49	Female	Healthy
HC-9	45	Male	Healthy

HC-10	49	Female	Healthy
