# Supplementary material for: Identification of Candidate mRNA and miRNA Molecules Associated with Tuberculosis Through Preliminary Analysis and Validation Using Clinical Samples
Source: Int J Mol Sci. 2026 Jun 7;27(12):5177. doi: 10.3390/ijms27125177 (PMC13299930; doi:10.3390/ijms27125177)
Supplement: Supplementary file 1 [file ijms-27-05177-s001.zip › Table S2.pdf]

Table S2. mRNA sequencing: raw data quality

Sample	Group	Raw reads (R1)	Raw bases (R1)	Raw Q20 (R1, %)	Raw Q30 (R1, %)	Raw reads (R2)	Raw bases (R2)	Raw Q20 (R2, %)	Raw Q30 (R2, %)
case-1	case	72843819	10744538797	98.2%	94.9%	72843819	10732758792	98.1%	94.7%
case-3	case	80873205	11871129463	98.3%	95.1%	80873205	11857923424	98.3%	95.0%
case-4	case	81469645	12005978031	98.3%	95.2%	81469645	11993456966	98.2%	94.9%
case-5	case	78675378	11595515874	98.2%	95.0%	78675378	11582128220	98.2%	94.8%
case-6	case	78460464	11572335001	98.2%	95.0%	78460464	11560188341	98.1%	94.8%
case-7	case	64434005	9412438277	98.2%	95.0%	64434005	9402914618	98.2%	94.8%
case-8	case	72827067	10750475904	98.1%	94.9%	72827067	10737632770	98.1%	94.8%
case-9	case	82637285	12155295497	98.3%	95.2%	82637285	12139729006	98.4%	95.5%
case-10	case	73142452	10812633784	98.3%	95.2%	73142452	10798527898	98.2%	95.0%
case-12	case	66033226	9602392965	98.2%	94.9%	66033226	9592444143	98.1%	94.6%
control-1	control	66649000	9814216784	98.2%	94.9%	66649000	9802758177	98.1%	94.7%
control-2	control	72720319	10740058309	98.2%	95.0%	72720319	10728278618	98.1%	94.5%
control-4	control	71398689	10551130877	98.2%	95.0%	71398689	10539450046	98.1%	94.7%
control-5	control	65588822	9696312083	98.2%	95.1%	65588822	9684823660	98.2%	94.7%
control-8	control	88598374	12863134105	98.2%	95.1%	88598374	12851240467	98.3%	95.1%
control-9	control	66283857	9798041330	98.3%	95.2%	66283857	9787481409	98.1%	94.7%
control-10	control	85177216	12592193303	98.1%	94.8%	85177216	12578135108	98.0%	94.5%
control-11	control	78628660	11649023359	98.2%	94.9%	78628660	11635755363	97.9%	94.1%
control-12	control	71044966	10514327986	98.2%	95.0%	71044966	10501319687	98.2%	95.0%
control-13	control	65670576	9711190743	98.2%	95.0%	65670576	9699782129	98.2%	94.9%
