# Supplementary material for: Identification of Candidate mRNA and miRNA Molecules Associated with Tuberculosis Through Preliminary Analysis and Validation Using Clinical Samples
Source: Int J Mol Sci. 2026 Jun 7;27(12):5177. doi: 10.3390/ijms27125177 (PMC13299930; doi:10.3390/ijms27125177)
Supplement: Supplementary file 1 [file ijms-27-05177-s001.zip › Table S3.pdf]

Table S3. mRNA sequencing: clean data quality

Sample	Group	Clean reads (R1)	Clean bases (R1)	Clean Q20 (R1, %)	Clean Q30 (R1, %)	Clean reads (R2)	Clean bases (R2)	Clean Q20 (R2, %)	Clean Q30 (R2, %)	Clean reads ratio (%)
case-1	case	72340149	10664727638	98.3%	95.1%	72340149	10650912635	98.4%	95.1%	99.3%
case-3	case	80428950	11800113877	98.4%	95.2%	80428950	11784469202	98.5%	95.3%	99.5%
case-4	case	80818183	11904247569	98.4%	95.3%	80818183	11888386983	98.5%	95.3%	99.2%
case-5	case	78243149	11525372472	98.4%	95.2%	78243149	11510050783	98.4%	95.1%	99.5%
case-6	case	77959273	11492848811	98.3%	95.2%	77959273	11477613317	98.4%	95.1%	99.4%
case-7	case	64009906	9345091639	98.3%	95.2%	64009906	9333205809	98.5%	95.2%	99.3%
case-8	case	72277937	10663433368	98.3%	95.1%	72277937	10648503211	98.4%	95.2%	99.2%
case-9	case	82173989	12081208848	98.4%	95.4%	82173989	12063538189	98.7%	95.8%	99.4%
case-10	case	72685905	10740663152	98.4%	95.3%	72685905	10724478488	98.5%	95.3%	99.4%
case-12	case	65575332	9529513913	98.3%	95.2%	65575332	9516910762	98.4%	95.0%	99.3%
control-1	control	66192123	9741927950	98.3%	95.1%	66192123	9728485913	98.4%	95.1%	99.3%
control-2	control	72247334	10666495675	98.3%	95.2%	72247334	10650870150	98.3%	94.9%	99.3%
control-4	control	70884333	10470476344	98.3%	95.2%	70884333	10455699301	98.4%	95.1%	99.3%
control-5	control	65208875	9635696337	98.4%	95.3%	65208875	9622685895	98.4%	95.0%	99.4%
control-8	control	88084053	12782017623	98.4%	95.3%	88084053	12765439299	98.5%	95.4%	99.4%
control-9	control	65821124	9727699907	98.4%	95.4%	65821124	9712644081	98.4%	95.0%	99.3%
control-1	contr	84565591	12494450702	98.3%	95.0%	84565591	12478523545	98.3%	94.8%	99.3%

0	ol									
control-11	control	78070047	11559777544	98.3%	95.2%	78070047	11544657146	98.2%	94.5%	99.3%
control-12	control	70609902	10444149266	98.3%	95.2%	70609902	10429713988	98.5%	95.3%	99.4%
control-13	control	65298094	9651319698	98.4%	95.2%	65298094	9638148977	98.5%	95.2%	99.4%
