# Supplementary material for: Identification of Candidate mRNA and miRNA Molecules Associated with Tuberculosis Through Preliminary Analysis and Validation Using Clinical Samples
Source: Int J Mol Sci. 2026 Jun 7;27(12):5177. doi: 10.3390/ijms27125177 (PMC13299930; doi:10.3390/ijms27125177)
Supplement: Supplementary file 1 [file ijms-27-05177-s001.zip › Table S4.pdf]

Table S4. Mapping rates.

Sample	Group	Mapping rate (%)
case-1	case	98.12
case-3	case	98.21
case-4	case	98.18
case-5	case	98.27
case-6	case	98.05
case-7	case	98.06
case-8	case	98.00
case-9	case	98.23
case-10	case	98.21
case-12	case	98.10
control-1	control	98.17
control-2	control	98.19
control-4	control	98.07
control-5	control	98.30
control-8	control	98.21
control-9	control	98.27
control-10	control	98.11
control-11	control	98.03
control-12	control	98.09
control-13	control	98.20
