# Supplementary material for: Identification of Candidate mRNA and miRNA Molecules Associated with Tuberculosis Through Preliminary Analysis and Validation Using Clinical Samples
Source: Int J Mol Sci. 2026 Jun 7;27(12):5177. doi: 10.3390/ijms27125177 (PMC13299930; doi:10.3390/ijms27125177)
Supplement: Supplementary file 1 [file ijms-27-05177-s001.zip › Table S5.pdf]

Table S5. Quality metrics of miRNA sequencing data

Sample	Group	Raw reads	Raw bases	Raw Q20 (%)	Raw Q30 (%)	Raw GC (%)	Clean reads	Clean bases	Clean Q20 (%)	Clean Q30 (%)	Clean GC (%)	Clean reads ratio (%)
case-1	case	11959086	259648725	98.2%	95.5%	49.5%	11780393	252786208	98.4%	95.7%	49.5%	98.5%
case-3	case	13436957	301645100	99.1%	97.4%	48.4%	12881884	275836823	99.3%	97.7%	47.7%	95.9%
case-4	case	13613550	295880686	99.0%	97.2%	46.8%	13308485	283242335	99.2%	97.4%	46.5%	97.8%
case-5	case	13468214	292266878	99.0%	97.1%	44.4%	13221786	282000874	99.1%	97.3%	44.2%	98.2%
case-6	case	12142174	264142475	99.2%	97.5%	44.9%	11883937	253126510	99.3%	97.7%	44.5%	97.9%
case-7	case	15292278	332605982	99.2%	97.6%	45.0%	15015016	320112674	99.3%	97.8%	44.7%	98.2%
case-8	case	11811163	261074690	99.1%	97.3%	45.3%	11464227	245126052	99.3%	97.5%	44.7%	97.1%
case-9	case	12600862	269349867	99.1%	97.5%	43.4%	12475754	264957369	99.3%	97.7%	43.3%	99.0%
case-10	case	11918222	254354611	99.1%	97.4%	43.5%	11804901	250656324	99.3%	97.6%	43.4%	99.0%
case-12	case	10027390	220808910	98.9%	96.9%	44.5%	9714630	206528689	99.0%	97.1%	43.9%	96.9%
control-1	control	15729191	340235239	99.1%	97.4%	44.8%	15426111	329155439	99.3%	97.6%	44.6%	98.1%
control-2	control	13656547	294519969	99.1%	97.5%	47.4%	13483058	289357043	99.3%	97.7%	47.4%	98.7%
control-4	control	12837269	277139911	99.2%	97.4%	44.3%	12594931	268185553	99.3%	97.6%	44.1%	98.1%
control-5	control	13290107	285729453	99.1%	97.4%	44.4%	13121851	279024296	99.3%	97.6%	44.3%	98.7%
control-8	control	11729707	256795228	99.1%	97.3%	49.9%	11540596	250009946	99.3%	97.5%	49.9%	98.4%
control-9	control	12412088	269207650	99.1%	97.3%	43.8%	12116807	257105961	99.3%	97.6%	43.3%	97.6%
control-10	control	11395319	248367857	99.1%	97.4%	46.1%	11165039	238244362	99.3%	97.5%	45.8%	98.0%
control-11	control	11577822	261435998	99.0%	97.2%	52.0%	11130126	241710742	99.2%	97.5%	51.8%	96.1%
contr	con	1396	31533	99.1%	97.3%	52.1%	13402	29079	99.3%	97.6%	51.9%	96.0%

ol-12	trol	0477	1434				060	6596				
contr	con	1192	26983	99.0%	97.1%	49.7%	11435	24717	99.2%	97.4%	49.3%	95.9%
ol-13	trol	3341	6134				757	8706				
