# Supplementary material for: Identification of Candidate mRNA and miRNA Molecules Associated with Tuberculosis Through Preliminary Analysis and Validation Using Clinical Samples
Source: Int J Mol Sci. 2026 Jun 7;27(12):5177. doi: 10.3390/ijms27125177 (PMC13299930; doi:10.3390/ijms27125177)
Supplement: Supplementary file 1 [file ijms-27-05177-s001.zip › Table S6.pdf]

TABLE S6 | 98 corresponding target genes.

NO.	ID
1	POGLUT3
2	NPEPPS
3	SLC52A2
4	CTDNEP1
5	TBC1D22B
6	PRKRA
7	CCNG1
8	PIP4K2A
9	PEG10
10	ADAM10
11	TFDP2
12	NR3C2
13	EIF5
14	AGO1
15	PTCD1
16	SLCO3A1
17	ATP5MF-PTCD1
18	NR2F2
19	TP53
20	EPHB2
21	SP1
22	ADIPOR2
23	PRKCA
24	GOSR1
25	MMP14
26	EGR2
27	VPS53
28	MYB
29	KIAA0232
30	RCC2
31	FAM104A
32	DDX6
33	SESN3
34	STX6
35	KLF4
36	DNMT1
37	NEURL4
38	CDK19
39	TNRC6B
40	RMND5A
41	NPTX1
42	TGFA

43	PTEN
44	LDLR
45	KLF6
46	TSPAN3
47	PAK4
48	ZNF584
49	CSGALNACT1
50	CCNL1
51	PODXL
52	KIT
53	POGZ
54	AAK1
55	TMEM178B
56	CCNT2
57	REL
58	COL4A5
59	BACE1
60	DNMT3A
61	VHL
62	HMGCR
63	PDGFC
64	PHACTR2
65	MAP2K6
66	NREP
67	ENPP2
68	DYNLT1
69	LAMA2
70	PPIC
71	ZBTB34
72	MXD1
73	PLAG1
74	ITGA6
75	NASP
76	PPM1D
77	OTUD4
78	NOVA1
79	NRP1
80	GTPBP2
81	KLHL15
82	MAK16
83	FAM117B
84	FBXO28
85	CREBRF
86	KRAS

87	MTHFD2
88	SIRT1
89	BCL2L11
90	AMOTL1
91	TNFAIP8
92	PRRX1
93	ONECUT2
94	REST
95	ZNF704
96	SERINC5
97	FOXP1
98	TBPL1
