# Supplementary material for: Identification of Candidate mRNA and miRNA Molecules Associated with Tuberculosis Through Preliminary Analysis and Validation Using Clinical Samples
Source: Int J Mol Sci. 2026 Jun 7;27(12):5177. doi: 10.3390/ijms27125177 (PMC13299930; doi:10.3390/ijms27125177)
Supplement: Supplementary file 1 [file ijms-27-05177-s001.zip › Table S7.pdf]

TABLE S7 | Clinical data of 10 patients.

TB group	Sex	Diagnostic basis
1	male	1. Main symptoms: cough, hemoptysis, the color red, about 200 milliliters in total, a little night sweats, and no fever, dyspnea, chest pain, chest tightness, etc.<br>2. Physical examination: acute appearance, emaciation, clear spirit, slight shortness of breath, no cyanosis, auscultation of breath sounds in both lungs, scattered wet rale can be heard in both lungs.<br>3. Chest CT: thoracic symmetry, trachea mediastinum centered; Multiple nodular and cable strip shadows were found in both upper lungs, and ground glass density shadows were diffusently distributed in both lungs. There were vesicular low-density shadows in the left upper lung, no enlarged lymph nodes in the mediastinum, a few calcified shadows in both upper lung lesions, and no abnormal bone.<br>4. Blood cell analysis (five classes) WBC 9.3*10~9/l, HCT 0.377l/l, MCH 31.2pg, NE% 91.9%; Three tests of tuberculosis antibody on protein chip, LAM: positive、38KD: positive. Blood gas analysis：PH 7.44, PCO_2 42mmHg, PO_2 60mmHg, SO_2 92%.
2	male	1. Main symptoms: cough, dry cough, accompanied by fatigue.<br>2. Physical examination: chronic appearance.<br>3. Auxiliary inspection:on lung CT, the thorax was symmetrical, and the trachea was centered. Patchy, cord and nodular high-density shadows were seen in each lobe of both lungs, and some lesions adhered to the pleura. Bronchial openings in each lobe were smooth, and calcified lymph nodes were seen in the mediastinum. After admission, whole-lung CT showed symmetrical thoracic cavity, centered trachea, patchy, banded and nodular high-density shadows in each lobe of both lungs, some lesions were adhered to the pleura, circular annular light transmission area was seen at the edge of the upper lobe of the left lung, bronchial openings of each lobe were smooth, and calcified lymph nodes were seen in the mediastinum. *Mycobacterium tuberculosis* DNA test: detected (very low concentration). Arterial blood gas: PH 7.44, HCO_3A 25.1mmol/L, BE 0.9mmol/L, SO2 96%, PCO_2 37mmHg, PO_2 79mmHg, Lac 0.9mmol/L; procalcitonin detection PCT 0.14ng/ml.

3	female	1. Main symptoms: cough, a small amount of yellow and white sticky phlegm, chest tightness, shortness of breath, aggravated after activity, accompanied by fatigue, weight loss, loss of appetite.<br>2. Physical examination: chronic appearance, percussion of both lungs showed clear sound, breath sounds of the right upper lung were weakened, and dry and wet rales and wheeze sounds could be heard.<br>3. Auxiliary inspection: all lung CT thoracic symmetry, tracheal center, under the left upper lobe, double lung lobe is patchy, a funicular, uneven nodular density increase, part of the lesions and pleural adhesion, can be seen in the lower lobe lesions irregular photic zone, right lung volume of the upper lobe narrow, consolidation of the plate shaped shadow, corresponding bronchus blocking, lung door, mediastinum, did not see swollen lymph nodes. Arterial blood gas: PH 7.41、HCO_3A 24.1mmol/L, BE -0.5mmol/L, SO_2 95%, PCO_2 38mmHg, PO_2 73mmHg, Lac1.0mmol/L; Blood cell analysis (five classes) HGB 108g/l, HCT 0.324l/l, PLT 344*10~9/l, MCV 80.2fl, MCH 26.7pg, NE% 70.2%, RDW-CV 14.6%， PCT 32.00%; Determination of C-reactive protein (CRP)(various immunological methods) CRP 25.59mg/L; Procalcitonin detection PCT 0.14ng/ml.
4	male	1. Main symptoms: cough, fatigue, fever once.<br>2. Past: clear history of TB exposure (mother of patient, currently in treatment).<br>3. Physical examination: chronic appearance, no wet or dry rales heard on auscultation of both lungs.<br>4. Chest CT: thoracic symmetry, mediastinal trachea centered; The left lower lobe of the lung showed multiple patchy shadows with increased density, unclear boundary, reduced mediastinal window, soft tissue density, and air-containing bronchial shadows; No abnormal density shadow was found in the other two lungs; The trachea and each lobe and segment of bronchus open patently; There were no enlarged lymph nodes in the mediastinum.<br>5. Tuberculin test: 20mm×20mm. C-reactive protein: 7.05mg/L. Calcitonin original: 0.07ng/ml. Blood sedimentation: 16mm/H.

5	male	1. Main symptoms: intermittent cough, initial hemoptysis, bright red, about 10-20 ml in total, slight chest tightness and chest pain when coughing, occasional wheezing, intermittent low fever in the afternoon, accompanied by fatigue, loss of appetite and other symptoms, regular anti-infection treatment is ineffective, no hemoptysis, still have low fever, cough a little yellow-white phlegm.<br>2. He had a long history of smoking and drinking, but did not quit.<br>3. Physical examination: chronic appearance, stable breathing, no cyanosis of lips, no anger of jugular veins, no swelling of superficial lymph nodes, coarse breath sounds in both lungs, and no dry or wet rals. Heart rate was 78 beats/min, rhythm was consistent, no murmur was heard in the auscultation area of each valve, the abdomen was soft and tender, the liver was not touched under the ribs, and there was no edema in both lower limbs.<br>4. Chest CT: thoracic symmetry, trachea centered; Scattered patchy and nodular shadows with increased density were seen in the upper and lower lobes of the left lung, and small cavity shadows were seen in the upper and lower lobes. Small heart shadow, no fluid density shadow in the chest.<br>5. Tuberculosis infected T cell spot test: tuberculosis specific antigen A 144 2.5X10 5, tuberculosis specific antigen B 18 2.5X10 5, positive.
6	male	1. Main symptoms: intermittent cough, a little yellow-white phlegm, slight fever, body temperature was not measured, accompanied by fatigue, night sweats, no obvious chest pain, chest tightness and dyspnea, intermittent anti-inflammatory treatment, symptoms did not improve.<br>2. Physical examination: chronic appearance, no cyanosis of lips, no swelling of superficial lymph nodes in the whole body, coarse breath sounds in both lungs, and no dry or wet rals.<br>3. Chest CT: thoracic symmetry, mediastinal trachea centered, bronchial opening of each lobe patency. The texture of both lungs was enhanced, and striped, patchy, nodular and clumpy high-density shadows and calcification shadows were seen in the right lobe, left upper lobe of the lung, and tongue lobe, with unclear boundaries. Cavity formation and irregular cave wall were seen in the lesions of the right upper lobe of the lung, which appeared to be nodular shadows. Slightly enlarged lymph nodes were seen in the mediastinum. Calcified lymph node shadows are seen in the mediastinum. Heart shadow is not big; Calcification was observed in the coronary artery shape area and thoracic aorta wall. Color Doppler ultrasound of liver, gallbladder and spleen indicates fatty liver.<br>4. T cell spot test for tuberculosis infection was positive. Xpert MTB/RIF to detect (a medium concentration).

7	femal e	1. Main symptoms: cough, whole mouth hemoptysis, volume of about 20 ml, accompanied by chest tightness.<br>2. Physical examination: chronic appearance.<br>3. Auxiliary examination: the lung CT: thoracic symmetry, centered the trachea, trachea right rear see limitations gas density with upper left lung is a little article nodular, lamellar high-density shadow and punctate calcification shadow, each lobe bronchus unobstructed, left lung door lymph nodes of calcification, inspection conclusion: tracheal diverticulum, consider the left upper lobe tuberculosis, left lung lymph node calcification.<br>4. T cell spot test for tuberculosis infection: positive.
8	male	1. Main symptoms: chest tightness with night sweats, fatigue, right chest pain, cough, expectoration.<br>2. Physical examination: chronic appearance, slightly distension of the right thoracic cavity, widening of the intercostal space, dullness in percussion of the right lower lung, weak breath sounds, wet rales can be heard on auscultation of the right upper lung.<br>3. Chest CT: thoracic symmetry, mediastinal trachea centered; Nodular and cable-like high-density shadows were seen in the upper lobe apex and posterior segments of the right lung, with blurred boundaries and reduced mediastinal window. Multiple nodular calcification density shadows were seen in the upper lobe apex and posterior segments of the right lung. Punctate calcification was observed in the posterior basal segment of the lower lobe of the left lung. In the posterior basal segment of the lower lobe of both lungs, there were cable strips with high density shadow with sharp border and indistinct mediastinal window. No abnormal density shadow was found in the other two lungs. The trachea and each lobe and segment of bronchus open patently; There were no enlarged lymph nodes in the mediastinum. The right interlobar pleura was thickened, and patchy water-like density shadow was observed in the right pleural cavity. Imaging diagnosis: secondary pulmonary tuberculosis in the upper lobe of the right lung with partial calcification; Calcification in the posterior basal segment of the lower lobe of the left lung; Cable strip shadow in posterior basal segment of lower lobe of both lungs; Thickening of the right interlobar pleura; Right pleural effusion.<br>4. Assay: C-reactive protein: 21.13mg/L. Chlorine tendency for 100.8 L. Esr: 54mm/H. Tuberculin test: 10mm×10mm. Pleural effusion assay: Lifantha test was positive, protein quantification was 38g/L, number of cells 2410×106/L, lobulated 30.0%, lymphatic 70%, acid-fast bacilli negative, glucose 3.8mmol/L，ADA 49.0U/L.

9	male	1. Main symptoms: intermittent cough, expectoration, occasional fever, accompanied by fatigue, loss of appetite, stomach discomfort, etc., fever occurred in the past one month, the temperature was up to 38.4℃, irregular, and fatigue increased, cough, weak expectoration, occasionally yellow sticky sputum, slightly peculiar smell.<br>2. He has a long history of smoking and has not quit. Medical history of diabetes and hypertension was denied.<br>3. Physical examination: body mass index of 14.69kg/m2, chronic pathological appearance, clear and clear, shortness of breath, no swelling of systemic superficial lymph nodes, drum sound in the left upper lung percussion, tubular breath sounds could be heard, slightly weak breath sounds in the left lower lung, scattered wet rale could be heard in the right lung. Heart rate 100 beats/min, rhythmic, no murmur in the auscultation area of each valve, scaphoid abdomen, soft abdomen, mild tenderness in the middle and upper abdomen, no muscle tension and rebound pain, large liver, and obvious edema in both lower limbs. Pathological reflex was not elicited.<br>4. Chest CT: double lung texture increased, lung air content increased, multiple cystic bright areas with clear boundaries; Large patchy soft tissue density shadows were seen in the upper lobe of the right lung with CT value of about 30HU. Multiple patchy high density shadows were seen in the other right lung, multiple cavities and cystic low density shadows were seen in the left lung, some bronchi of the left lung were unclear, and calcification shadows were seen in the upper lobe of the left lung. No enlarged lymph nodes were observed in the mediastinum, and strips of fluid density were seen in the pericardium and the dorsal thorax on both sides.<br>5. Tuberculosis smear test (fluorescence)(3+)10-99 bar/per view. Xpert MTB/RIF to detect (a high concentration).

10	male	1. Main symptoms: low fever, no obvious regularity, accompanied by left seasonal rib pain, cough, occasional sputum, gradually chest tightness, shortness of breath, accompanied by fatigue, loss of appetite, etc.<br>2. He had a history of occasional drinking and denied smoking. Medical history of hypertension and diabetes was denied.<br>3. Physical examination: chronic pathological appearance, emasculation, clear breath, stable breathing, no swelling of superficial lymph nodes, dullness in percussion below the fifth anterior rib of the left midclavicular line, weakened breath sounds on auscultation, no pleural friction. The abdomen is soft, without tenderness, the liver and spleen are not large, and the limbs are not edema.<br>4. Chest CT: symmetrical thoracic cavity, centered trachea: patchy and nodular shadows with increased density were seen in the upper lobe of the left lung, lung tissue was not distended, and a few ground-glass shadows were seen in the lower lobe of the right lung. The left pleura was thickened with a pleural effusion in which a drainage tube was seen, and a small pleural effusion was observed on the right side. Heart shadow is not big. The lymph nodes are not large.<br>5. Blood spot test for tuberculosis specific antigen A 60 2.5X10E5; Tuberculosis specific antigen B 25 2.5X10E5, Positive. Full set of pleural effusion: Adenosine deaminase assay 45.0U/L, carcinoembryonic antigen negative, acid-fast bacilli negative, tuberculosis antibody negative, protein quantification 29g/L, determination of glucose 4.6mmol/L, cell population 720X10~6/L, classification:segmented 30.0%, lymphatic 70.0%. Li Fan it test positive. Hydrothorax xpert MTB/RIF not checked out. Blood tuberculosis antibody weak positive, erythrocyte sedimentation rate 45mm/h.
