# Supplementary material for: Identification of Candidate mRNA and miRNA Molecules Associated with Tuberculosis Through Preliminary Analysis and Validation Using Clinical Samples
Source: Int J Mol Sci. 2026 Jun 7;27(12):5177. doi: 10.3390/ijms27125177 (PMC13299930; doi:10.3390/ijms27125177)
Supplement: Supplementary file 1 [file ijms-27-05177-s001.zip › Table S8.pdf]

TABLE S8 | The differentially expressed genes.

ID	log2FoldChange	P Value	padj	type
hsa-let-7a-3p	1.167970513	0.000443136	0.010886371	Up
hsa-miR-643	1.287892082	0.008734908	0.072006124	Up
hsa-miR-548ae-3p	1.265243311	0.022472623	0.128390104	Up
hsa-miR-98-3p	1.309285752	0.00712462	0.064825242	Up
hsa-miR-3194-3p	1.034793094	0.010048387	0.077954329	Up
hsa-miR-199b-5p	1.314270457	4.49464E-05	0.003312549	Up
hsa-miR-135a-5p	1.240385611	0.001090237	0.017627321	Up
hsa-miR-4772-3p	1.234888024	0.008116475	0.070951907	Up
hsa-miR-24-1-5p	1.206182472	0.003123142	0.034354557	Up
hsa-miR-29b-3p	1.363314824	3.22354E-06	0.000395958	Up
hsa-miR-570-3p	1.411394834	0.00015033	0.006517252	Up
hsa-miR-581	1.098962929	3.02074E-09	2.22629E-06	Up
hsa-miR-2115-3p	1.484292056	0.021124792	0.123563269	Up
hsa-miR-149-5p	1.688408489	0.005616362	0.053756612	Up
hsa-miR-2115-5p	1.052519406	0.00259146	0.031335816	Up
hsa-miR-607	1.362326045	0.000186402	0.006868924	Up
hsa-miR-199a-3p	1.035568461	0.015364973	0.097475195	Up
hsa-miR-5582-3p	1.183389626	0.016056181	0.099440378	Up
hsa-miR-26a-2-3p	1.477841619	1.81623E-05	0.0016732	Up
hsa-miR-548ar-3p	2.36452411	0.000824924	0.015686057	Up
hsa-miR-152-3p	1.037022462	0.000535453	0.012332157	Up
hsa-miR-26a-1-3p	1.348078353	0.005426924	0.052626886	Up
hsa-miR-1284	1.06901798	0.000727748	0.015324292	Up
hsa-miR-145-3p	1.508051067	9.21666E-07	0.000226423	Up
hsa-miR-29c-3p	1.349250874	5.25081E-05	0.00351804	Up
hsa-miR-338-3p	1.639975044	0.017345096	0.104781443	Up
hsa-miR-9-5p	1.813071889	6.32802E-05	0.003886458	Up
hsa-miR-3140-3p	1.232874677	0.018109936	0.107637281	Up
hsa-miR-9903	1.085437866	0.000171154	0.006638981	Up
hsa-miR-143-5p	1.036023102	0.002100218	0.027640372	Up
hsa-miR-199b-3p	1.035568461	0.015364973	0.097475195	Up
hsa-miR-212-5p	1.092972414	0.001272488	0.019953691	Up
hsa-miR-10527-5p	-1.538582828	0.000159203	0.006518468	Down
hsa-miR-1306-3p	-1.060528162	0.008337741	0.071452502	Down
hsa-miR-4508	-1.137698159	0.002048067	0.027444103	Down
hsa-miR-1287-5p	-1.201081375	0.003022862	0.034354557	Down
hsa-miR-5010-5p	-1.193060651	0.026088518	0.137337414	Down
hsa-miR-6780a-5p	-1.823363772	0.001520565	0.021973648	Down
hsa-miR-150-5p	-1.249629513	0.001057616	0.017627321	Down
hsa-miR-4446-3p	-1.798425429	0.004661709	0.046428104	Down
hsa-miR-877-5p	-1.001192459	0.000866601	0.015955985	Down
hsa-miR-210-5p	-1.00516185	0.002939105	0.034354557	Down

hsa-miR-320e	-1.176091859	0.000766057	0.015682893	Down
hsa-miR-1270	-1.348036662	0.000789084	0.015686057	Down
hsa-miR-4429	-1.178011926	0.043317815	0.192320659	Down
hsa-miR-122-5p	-2.938073952	2.27606E-05	0.001863837	Down
hsa-miR-7109-3p	-1.145032356	0.0155773	0.097475195	Down
hsa-miR-432-5p	-1.29051807	0.025796602	0.136777666	Down
hsa-miR-574-5p	-1.62571666	0.000949881	0.016668158	Down
AAK1	2.494081353	0.026885414	0.196573621	Up
AANAT	1.092047065	0.029828615	0.208773865	Up
AASDH	2.579319115	0.000486888	0.013218211	Up
AATK	1.473384533	0.001331404	0.027419702	Up
ABAT	1.698926123	0.006043586	0.074986909	Up
ABCA1	1.18667019	1.27998E-06	0.000117949	Up
ABL2	1.496042003	0.011570069	0.115715601	Up
ACE2	1.893104501	0.011306999	0.113877209	Up
ACHE	1.258590864	0.01804787	0.153377244	Up
ACP5	1.538838503	0.024718606	0.186264327	Up
ACSL1	1.03391655	0.001390316	0.028077726	Up
ACSL4	1.192199943	3.01821E-09	3.9692E-07	Up
ADAM15	1.722304814	0.041956011	0.258411929	Up
ADGRG3	1.277218195	2.39954E-05	0.001391484	Up
ADM	1.59362475	5.23207E-06	0.000397218	Up
AFF1	1.484817418	1.70716E-05	0.001042981	Up
AFF2	2.731106238	0.007068518	0.08352822	Up
AGFG1	1.317206849	0.010052607	0.106269028	Up
AIG1	1.151651993	0.039970518	0.249864044	Up
AIM2	2.133822942	9.41956E-09	1.20605E-06	Up
ALOX5AP	1.386908938	8.97856E-05	0.003893978	Up
ALPK1	1.346012012	8.25184E-07	8.0841E-05	Up
ALPL	1.498654817	0.003383825	0.051723999	Up
AMD1	1.421863704	0.029450846	0.207208123	Up
AMFR	4.096291529	0.000168447	0.006217796	Up
ANGEL1	2.070772095	0.003819918	0.056027871	Up
ANKHD1-EIF4EBP3	1.306409101	0.000427324	0.012065516	Up
ANKRD11	2.453544974	0.012242282	0.119843387	Up
ANKRD13D	1.048724412	0.00249285	0.042139484	Up
ANKRD22	4.599023515	1.59792E-13	2.55109E-11	Up
ANO2	2.115838494	0.007019257	0.083144216	Up
ANOS1	3.021580878	0.00261579	0.043553471	Up
ANXA3	1.897537989	6.29025E-05	0.003009006	Up
AP3B2	1.783425737	0.006506843	0.078688584	Up
AP5B1	1.113094581	1.16838E-06	0.000109824	Up
APOBEC3A_B	1.389188432	0.000636131	0.016129867	Up
APOBEC3B	1.518028212	0.000811506	0.019530002	Up

APOL1	1.155157746	0.000436097	0.012197813	Up
APOL2	1.701665629	6.80624E-06	0.000496331	Up
APOL4	1.616808427	0.000449217	0.012457016	Up
APOL6	1.341528952	1.4504E-06	0.00012995	Up
AQP1	1.02480899	0.008204702	0.09247572	Up
AQP10	1.545600028	0.002584823	0.043173553	Up
AQP9	1.043106102	0.000275043	0.008833028	Up
ARAP1	1.100503103	7.43678E-05	0.003374383	Up
ARF1	2.184097233	0.002243884	0.039089121	Up
ARFGAP2	2.043088113	0.003262335	0.050503702	Up
ARFIP1	1.001982209	0.043440681	0.263861159	Up
ARHGAP19	1.714486036	0.043806157	0.264878409	Up
ARHGAP45	2.372859522	0.00247944	0.041967662	Up
ARHGDIB	1.135214555	0.037191046	0.239314421	Up
ARHGEF2	1.498614842	0.01253499	0.121444121	Up
ARMCX6	1.418619316	0.036701119	0.237603771	Up
ARRB2	1.208032911	0.003321841	0.051074468	Up
ARV1	1.824100724	0.034868712	0.229276553	Up
ASAH1	2.216297191	0.000304939	0.009471664	Up
ASAP1	1.173564182	0.006043942	0.074986909	Up
ASPH	2.35280681	0.002987881	0.047681967	Up
ATF2	1.513636701	0.003026547	0.048154962	Up
ATF3	1.578944648	0.046729449	0.27471874	Up
ATP6V0E2	2.687334139	0.007179111	0.084392533	Up
ATPAF1	1.877414868	0.005558195	0.071471722	Up
B3GNT2	1.404455666	0.018042908	0.153368668	Up
B4GALT5	1.106941419	1.9311E-05	0.001156125	Up
BATF2	2.610721192	6.31578E-05	0.003017496	Up
BCL2A1	1.314491125	1.99353E-05	0.001182555	Up
BCL6	1.250772741	4.22408E-05	0.00219669	Up
BEND7	1.687282086	0.001690703	0.032183925	Up
BHLHA15	1.599922651	0.03441485	0.227485751	Up
BMX	3.171252006	9.03091E-07	8.76013E-05	Up
BRMS1	1.387603701	3.58657E-06	0.000284542	Up
BSND	1.099971143	0.022433268	0.175255887	Up
BTF3	1.375663003	0.007517407	0.087239039	Up
BTNL8	1.195721427	5.35277E-06	0.000404797	Up
C11orf1	1.567565869	0.022989957	0.177703804	Up
C19orf38	1.077671048	1.1379E-05	0.00074697	Up
C1orf43	1.218926425	0.001425154	0.028514114	Up
C1QA	1.51872246	0.024001289	0.182753684	Up
C3orf86	1.206692112	0.005778356	0.072924952	Up
C4orf33	1.765528548	0.00073214	0.017942757	Up
C6orf89	1.765981179	0.000156261	0.005885597	Up

CA12	1.283900622	0.002179471	0.038328466	Up
CA4	1.640743328	1.4843E-06	0.000132681	Up
CACNA1E	2.403661228	1.15348E-06	0.000109109	Up
CALHM6	1.403390532	0.001176544	0.025273537	Up
CAMKK1	1.234410987	0.017211808	0.148847991	Up
CAPG	1.14320598	0.036765198	0.23778993	Up
CAPN1	1.905459797	0.001154514	0.024938406	Up
CAPN11	1.867054981	0.002910229	0.046789201	Up
CAPN13	1.927673371	0.026010916	0.193054044	Up
CAPNS2	1.331716224	1.3874E-05	0.00087948	Up
CARD16	1.400609869	9.93043E-05	0.004199334	Up
CARD17	2.901237586	3.44032E-07	3.71775E-05	Up
CARM1	1.237691455	0.031184899	0.214430728	Up
CASP1	1.34945661	0.001937757	0.035441704	Up
CASP4	1.422232644	0.00010621	0.004397475	Up
CASP7	1.98432249	0.028266697	0.202872417	Up
CASP8	1.12213283	0.016393729	0.144085803	Up
CCDC125	1.149777516	0.00854245	0.095203774	Up
CCDC175	1.585021774	0.017887775	0.152441299	Up
CCNI	1.139257702	8.85876E-06	0.000611564	Up
CD177	1.601843278	0.028135012	0.202479169	Up
CD274	2.813544018	4.52824E-08	5.54173E-06	Up
CD300LD	2.224720957	0.003488692	0.05282741	Up
CD46	1.030117302	1.48435E-05	0.000931804	Up
CD63	1.810580992	1.96011E-05	0.001169886	Up
CD74	1.115494719	0.011964618	0.118198966	Up
CDA	1.148365322	6.48146E-07	6.53112E-05	Up
CDC42EP1	1.194168996	0.009423966	0.101275004	Up
CDC6	1.200911154	0.001160927	0.025021205	Up
CDCA5	1.462295637	0.001498071	0.029471427	Up
CDH23	1.343963644	9.15723E-05	0.003955396	Up
CEACAM1	1.27104113	0.000129273	0.005086442	Up
CEACAM4	1.111620846	2.61091E-05	0.001491755	Up
CEBPB	1.146350125	5.39497E-06	0.000406403	Up
CEBPD	1.173480756	2.8229E-06	0.000234005	Up
CELF2	1.042322604	0.008628852	0.095617338	Up
CENPA	1.671392637	0.016141992	0.142681377	Up
CEP55	1.320064347	0.034393363	0.227485751	Up
CERS1	1.006936334	0.013165489	0.125461835	Up
CETP	1.397320498	2.38712E-07	2.63841E-05	Up
CFD	3.354611512	0.016129958	0.142640012	Up
CHCHD5	1.149331955	0.040721712	0.252970184	Up
CHFR	1.920239263	0.000311438	0.009547993	Up
CHIT1	1.548590281	0.041161589	0.254886485	Up

CHM	1.915760903	0.040659898	0.252687999	Up
CKAP2L	1.029675242	0.008612776	0.095575696	Up
CLASP1	1.190921556	0.010433291	0.108718643	Up
CLEC4C	2.423746763	0.006085807	0.075310647	Up
CLEC4D	1.704508021	6.11669E-08	7.36947E-06	Up
CLEC4E	1.317149025	1.36078E-07	1.5554E-05	Up
CLEC6A	1.021487965	0.000159567	0.00596556	Up
CLIP1	1.591777351	0.034025277	0.226205863	Up
CLN5	3.349934337	5.27052E-05	0.002601397	Up
CLOCK	1.086180027	0.024857742	0.187059244	Up
CLPTM1	1.8809062	0.004118796	0.059053682	Up
CLTA	2.150618669	0.007701748	0.088714168	Up
CLUAP1	6.72990813	2.6639E-11	3.75803E-09	Up
CMPK2	1.070943491	0.03977505	0.249204307	Up
CNBP	1.126775459	0.00024953	0.008276765	Up
CNDP1	2.009926607	0.001017802	0.022970114	Up
CNGB1	1.46550276	0.008020168	0.09119063	Up
CNNM2	7.131334855	2.91452E-12	4.15695E-10	Up
COA1	1.344482725	0.02051544	0.165984668	Up
COPA	1.196592959	0.012020788	0.118572706	Up
COPE	1.446473974	0.021814678	0.172299625	Up
COPS3	1.67851645	0.042835624	0.261660845	Up
CPA3	1.332563296	0.026659654	0.195630062	Up
CPNE5	1.720386242	0.004531144	0.062691054	Up
CREG2	1.14930658	0.004705028	0.064271683	Up
CRISPLD2	1.248113028	4.38707E-05	0.002263251	Up
CRYBG1	2.072855501	0.001371301	0.027838627	Up
CSAD	1.839896429	0.012526275	0.121398161	Up
CSGALNACT1	1.914176985	0.019506938	0.160775795	Up
CTDP1	2.157406289	0.002375266	0.040737604	Up
CXCL16	1.413328523	1.30719E-06	0.000119605	Up
CXorf40A	3.538309211	1.11784E-07	1.29067E-05	Up
CXXC5	1.251383962	0.017548242	0.150615941	Up
CYBC1	1.000271486	0.004594528	0.063231003	Up
CYREN	1.562061278	0.026918819	0.196763734	Up
DAAM2	1.418796559	0.013074689	0.12487849	Up
DAP	1.609722361	0.034786032	0.229082351	Up
DBNL	1.098331592	0.002197063	0.038480845	Up
DCTN4	1.335755773	0.028431522	0.203430549	Up
DDIAS	1.766350807	0.000494783	0.013392279	Up
DFFB	1.549585739	6.25845E-05	0.003001196	Up
DHRS12	1.219439927	0.000309545	0.009530792	Up
DHRS7B	1.014419028	0.014019483	0.130365738	Up
DHRS9	1.003049475	9.49554E-05	0.004043682	Up

DIAPH2	1.133497127	0.029701107	0.208311219	Up
DIDO1	1.246346576	0.048390678	0.280907409	Up
DIS3L	1.993318828	0.022001261	0.173224984	Up
DNAJC25-GNG10	4.119861813	2.38195E-05	0.001383349	Up
DNASE1L1	1.589097175	0.028950853	0.205441435	Up
DNMT3A	1.043820128	0.043709217	0.264746145	Up
DOK3	1.336350593	0.002812194	0.04580145	Up
DPH7	2.046057998	0.03614698	0.235249469	Up
DSC2	1.273453352	6.33859E-05	0.003023754	Up
DTNB	2.449162276	0.000218156	0.007456704	Up
DUSP1	1.10346501	1.53203E-05	0.000955546	Up
DUSP3	1.076869149	6.53052E-07	6.56351E-05	Up
DYRK1A	1.473333727	0.037313294	0.239822605	Up
DYSF	1.962175313	2.35744E-08	2.9034E-06	Up
E2F1	1.30243086	0.005421468	0.07024911	Up
E2F8	1.02802747	0.020288891	0.164529169	Up
EBF1	3.246085887	6.47796E-05	0.003064787	Up
ECE1	1.880971271	0.000444007	0.01236677	Up
ECHDC3	1.447251002	5.77291E-06	0.000431575	Up
EFCAB2	1.120720158	9.74026E-05	0.004125254	Up
EGLN2	1.221749645	0.024952177	0.187491708	Up
EIF2AK2	2.031496238	6.73022E-05	0.003149563	Up
EIF4G1	3.117558085	4.07281E-05	0.002132314	Up
EIF4G3	1.780486425	0.04859244	0.281364735	Up
EIF5	1.550223837	0.001095924	0.024164126	Up
ELF2	1.167261044	0.002344813	0.040340139	Up
EMC9	2.111797122	0.012443777	0.120900659	Up
ENC1	2.810007506	5.99361E-05	0.002902896	Up
ENTPD1	1.54291579	0.019074566	0.158627605	Up
EPB41L3	2.54020769	0.000942095	0.021690538	Up
EPHB4	1.091354746	0.018650246	0.156305095	Up
EPN1	1.824793849	0.003705102	0.055049532	Up
EPSTI1	2.710202262	1.19404E-05	0.00077723	Up
ERC1	2.132550758	0.004509931	0.062486708	Up
EREG	1.642858212	0.002794737	0.045620238	Up
ERI1	1.347428979	0.013476141	0.127358559	Up
ERLIN1	2.502863886	0.007367302	0.085893547	Up
ERV3-1-ZNF117	1.390147244	0.011007276	0.11218064	Up
ETV7	3.329111596	7.43726E-07	7.37924E-05	Up
EVI2A	1.151170506	0.044187538	0.266194707	Up
EXOC3L1	1.370414593	0.004157493	0.059297774	Up
EXOC6	1.02222058	0.000369349	0.010847005	Up
F5	1.23697902	7.21669E-06	0.000521362	Up
FAM222B	1.964191132	0.029073585	0.205807721	Up

FAM49B	1.465692563	5.20304E-06	0.000396322	Up
FAS	1.107836768	1.26775E-06	0.000117101	Up
FBXL12	1.300954257	0.026068348	0.193221547	Up
FBXL5	1.60431998	0.027142984	0.197720746	Up
FBXO6	1.5547347	5.1737E-06	0.000395106	Up
FCAR	1.434867945	3.14642E-06	0.000254834	Up
FCER1G	1.024494407	6.98088E-05	0.003239511	Up
FCGR1A	2.900710799	3.03781E-12	4.31692E-10	Up
FCGR1B	2.294796792	6.02013E-10	8.19477E-08	Up
FCGR3A	1.543178737	0.028363705	0.203288776	Up
FCGR3B	1.248530144	0.045493708	0.270764489	Up
FDPS	1.056301664	0.020203963	0.16401187	Up
FFAR2	1.052405896	0.000384717	0.011196607	Up
FFAR3	1.071517178	0.042464754	0.260050534	Up
FGD4	1.220130063	0.014924751	0.135503323	Up
FGF13	1.999245226	0.005332999	0.06949738	Up
FKBP5	1.308873037	5.5542E-08	6.71263E-06	Up
FLNB	2.688996731	0.008983736	0.09812051	Up
FNDC3B	1.106883293	0.000308909	0.009530792	Up
FOXD4L1	1.38190386	4.07419E-07	4.34077E-05	Up
FOXJ3	1.212502214	0.043939553	0.265395446	Up
FOXO3B	1.045479539	0.046900052	0.275471235	Up
FOXO4	2.184345881	0.026025968	0.193054961	Up
FPR1	1.084015806	0.000245097	0.008175886	Up
FPR2	1.043865985	0.000576751	0.015057238	Up
FRMD3	1.02873696	0.01459272	0.133552015	Up
FUT8	1.857316228	0.002067749	0.036932924	Up
FYB1	1.187011149	9.03223E-07	8.76013E-05	Up
G0S2	2.058626584	2.31715E-06	0.000199764	Up
G6PD	2.651991676	0.000198942	0.006983523	Up
GAB2	3.031368969	5.17156E-05	0.002562331	Up
GADD45G	1.463337903	0.002207563	0.038577654	Up
GAPVD1	2.229315478	0.001402597	0.028273905	Up
GAS2L2	1.018083594	0.009994444	0.10596733	Up
GAS7	1.553686504	0.001145625	0.024808792	Up
GASK1B	1.149451364	0.000173037	0.006344947	Up
GBP1	2.450670887	3.63973E-09	4.77038E-07	Up
GBP2	1.392890227	7.22838E-08	8.62846E-06	Up
GBP4	1.298638073	5.00636E-06	0.000384598	Up
GBP5	2.676108774	1.80495E-10	2.50083E-08	Up
GBP6	3.058597614	9.30885E-10	1.24961E-07	Up
GCA	2.752226973	2.50362E-07	2.75931E-05	Up
GET1	3.846064112	1.36616E-05	0.000870282	Up
GIMAP6	1.262653125	0.000121694	0.00485255	Up

GK	1.290614636	2.96304E-05	0.001658744	Up
GLCE	1.665183462	0.003562966	0.053472059	Up
GLT1D1	1.021240825	0.001100859	0.024215686	Up
GLUL	1.029066073	0.000869881	0.020502458	Up
GP1BB	1.074226912	0.001527207	0.029893026	Up
GPAT3	1.162930509	0.000186504	0.006678291	Up
GPR17	1.253784446	0.005054831	0.067296561	Up
GPR42	1.553587156	0.003279454	0.050653592	Up
GPR84	1.214681399	0.004064495	0.058552572	Up
GPR87	1.834345676	0.000159615	0.00596556	Up
GRAMD1A	1.716513077	0.045393581	0.270389065	Up
GRAMD1B	2.604302476	0.000186831	0.006678291	Up
GRINA	2.03202295	0.00031927	0.009722201	Up
GSDMC	1.466475481	0.021607159	0.171246116	Up
GSN	1.451654655	0.012927593	0.124017298	Up
GUCY2C	1.272378166	1.09222E-05	0.000725874	Up
GUK1	3.277100942	5.07511E-05	0.002524217	Up
GYG1	1.263501856	3.83359E-05	0.002020829	Up
H1-5	1.449850786	0.000105503	0.004372843	Up
H1-6	1.231318795	0.000321872	0.009791816	Up
H2AC12	1.52753593	8.96539E-05	0.003893978	Up
H2AC13	1.151021714	0.003159978	0.049451929	Up
H2AC16	1.468458068	0.000363166	0.010730414	Up
H2AC4	1.283584156	0.000372036	0.010892943	Up
H2AC7	1.6973161	0.02097513	0.168293254	Up
H2BC12	1.012799067	9.32768E-06	0.000639342	Up
H2BC13	1.170895804	0.002267905	0.03941907	Up
H2BC14	1.497447013	0.001484246	0.029303481	Up
H2BC17	1.290652501	4.15106E-05	0.002161617	Up
H2BC18	1.026515228	0.012430163	0.120855825	Up
H2BC3	1.385909881	0.001515927	0.029732242	Up
H3C12	1.664713209	0.000437429	0.012226266	Up
H3C14	1.229934334	0.000851575	0.020205268	Up
H3C2	1.581600859	0.000286065	0.00906516	Up
H3C3	1.839565367	2.27156E-05	0.001323202	Up
H3C8	1.833063164	0.000138315	0.005371283	Up
H6PD	1.771805749	0.016872082	0.146991341	Up
HADHB	1.096501625	0.008517974	0.09506755	Up
HAUS4	1.428267882	0.000304392	0.009469834	Up
HDLBP	1.73822195	0.000199272	0.006983523	Up
HELZ2	1.114837875	7.13656E-06	0.000517501	Up
HK3	1.007081071	8.78136E-05	0.003845068	Up
HLA-B	1.025887626	4.96396E-07	5.12172E-05	Up
HLA-F	1.446046944	1.09936E-06	0.000104278	Up

HORMAD1	1.3245532	0.000680598	0.016992223	Up
HOXA9	1.155004335	0.015106691	0.136579837	Up
HSPH1	1.358995415	0.03870016	0.244746691	Up
HTATIP2	1.324669901	0.005076049	0.067486399	Up
ICAM1	1.253829147	1.19831E-09	1.59754E-07	Up
IFI35	1.24284259	0.000257091	0.008466752	Up
IFI44	1.524100301	0.008233734	0.092722122	Up
IFI44L	1.700218386	0.01098611	0.112159511	Up
IFI6	1.320808769	0.009150923	0.099192524	Up
IFIT1	1.105367064	0.049588824	0.284504072	Up
IFIT2	1.167039213	0.005850771	0.073501722	Up
IFIT3	1.544275963	0.001230485	0.025955174	Up
IFITM1	1.363269758	1.37762E-05	0.000876145	Up
IFITM3	2.144783637	1.2276E-06	0.000114208	Up
IFNGR2	2.661769419	3.80838E-07	4.08138E-05	Up
IGFLR1	1.063827572	2.51546E-06	0.000213539	Up
IGLL5	1.104645838	0.007368314	0.085893547	Up
IL17REL	1.185527028	2.00209E-05	0.001185817	Up
IL18R1	1.450450095	0.000871183	0.020520671	Up
IL18RAP	1.012965401	0.015503198	0.138998511	Up
IL1B	1.008456295	0.001805182	0.033612243	Up
IL1RAP	1.287269307	0.001008889	0.022848707	Up
IL2RB	4.062558321	2.31064E-05	0.001343945	Up
IP6K1	1.024437621	0.00419461	0.059564377	Up
IQSEC1	1.486274742	0.00193628	0.035441704	Up
IRF1	1.171226772	9.95345E-07	9.5429E-05	Up
IRF7	1.052065795	0.004017254	0.058152756	Up
ISG15	1.165895664	0.027541001	0.19993509	Up
ISG20	1.568341019	0.040627831	0.252629699	Up
IST1	2.295377574	0.028155063	0.202479169	Up
ISY1-RAB43	1.98831589	0.045902135	0.272247869	Up
ITCH	1.497521705	0.01040009	0.108576826	Up
ITGA2B	1.407674154	0.000787292	0.019054179	Up
ITPRIP	1.330330078	0.01220603	0.119639448	Up
JADE1	3.263839571	0.001475673	0.029193649	Up
JAK2	1.018937596	7.95153E-06	0.000561894	Up
JAML	1.307696586	0.000251749	0.008333288	Up
JCHAIN	1.427513452	0.00118745	0.02539532	Up
KCNE1	1.187332971	0.008497884	0.094897932	Up
KCNH7	1.279102456	0.000134571	0.005246922	Up
KCNJ15	1.427416314	4.6562E-05	0.002364777	Up
KCNJ2	1.377434205	3.06563E-06	0.000249332	Up
KDM2B	1.259906185	0.011017096	0.11218064	Up
KIAA0040	1.227919565	0.002567734	0.042959256	Up

KIAA0513	1.034456328	0.025977897	0.192935834	Up
KIF22	1.910568392	0.024406212	0.184677	Up
KIFC1	1.002872303	0.006292118	0.076955463	Up
KL	1.938351336	6.99492E-05	0.00324215	Up
KLF5	1.53356363	2.99829E-06	0.000244881	Up
KLHDC8B	1.017452285	3.59803E-05	0.00193533	Up
KLHL2	1.546769559	0.013787251	0.128916945	Up
KPTN	1.427773126	3.47861E-06	0.00027711	Up
KREMEN1	2.438481305	2.66283E-10	3.67631E-08	Up
L3MBTL3	1.88038605	0.017822316	0.152126896	Up
LACTB	1.537824442	0.024166434	0.183421426	Up
LAMP3	1.41404017	0.002434891	0.041398301	Up
LANCL1	1.366435047	0.028096548	0.202415148	Up
LAP3	1.157362901	0.000122783	0.004880513	Up
LASP1	1.353099037	0.030775202	0.212563082	Up
LHFPL2	1.572226145	6.68954E-06	0.00048874	Up
LILRA5	1.628897599	0.00029827	0.009339634	Up
LILRA6	1.249553828	0.001224174	0.025881098	Up
LILRB3	1.037982213	0.01773777	0.151650373	Up
LILRB4	2.458602597	0.00411711	0.059053682	Up
LIMK1	1.151013381	0.00024536	0.008177596	Up
LIMK2	1.177555449	1.6833E-05	0.001031136	Up
LIPA	1.178609564	0.001002616	0.022759804	Up
LIPM	4.500606387	1.60733E-07	1.82328E-05	Up
LIPN	1.258776272	0.000353507	0.010509058	Up
LMNB1	1.181580182	3.11394E-06	0.000252731	Up
LMTK2	1.133192329	2.19376E-08	2.71907E-06	Up
LOC100129484	1.104873141	3.63321E-06	0.000287654	Up
LPCAT2	1.145760131	2.52085E-05	0.001448837	Up
LRG1	1.18677313	0.00011055	0.004526969	Up
LRRC61	1.097043058	0.000515385	0.013817799	Up
LRRK2	1.109100574	2.97315E-05	0.001662007	Up
LRWD1	1.568890067	0.045423787	0.270485927	Up
LY6G6F	1.04100364	0.002900499	0.046727959	Up
LY96	1.458550028	0.005400687	0.070102161	Up
LYPD8	1.092224438	7.97231E-07	7.86987E-05	Up
LYPLA1	1.466603288	4.68386E-05	0.002375301	Up
LYSMD2	1.762942287	0.006012151	0.07482881	Up
MAFF	1.881879079	0.016751893	0.146409129	Up
MAGED2	2.310456701	0.023441186	0.180002912	Up
MALL	1.572128113	0.009146534	0.099191404	Up
MAOB	2.468076524	2.45392E-05	0.001416667	Up
MAP2K7	1.682263679	0.041117795	0.25473728	Up
MAP3K2	1.05083976	0.002099347	0.037309623	Up

MAPK14	1.14443836	1.32368E-05	0.000850202	Up
MARK2	1.229527348	0.000111558	0.004553881	Up
MBOAT7	1.174724791	0.010119029	0.10667601	Up
MCEMP1	1.677545577	3.93646E-05	0.002066508	Up
MCL1	1.806966947	0.000310196	0.009541204	Up
MCPH1	3.511586122	3.55279E-05	0.001919647	Up
MCRIP1	2.38843882	0.001988985	0.036016468	Up
MCTP1	1.096782626	9.37546E-06	0.000641483	Up
MDM4	1.163357961	0.001368238	0.027838627	Up
MED24	2.704244434	0.000685761	0.01708677	Up
MEF2A	1.380004835	0.011776985	0.116991271	Up
MEF2B	2.06392898	0.007419676	0.086362541	Up
MEPCE	1.528152322	0.006289843	0.076951894	Up
METTL7B	2.382802681	6.86563E-05	0.003193671	Up
MFAP3	1.71511742	0.006045201	0.074986909	Up
MFSD14B	1.192980767	1.16916E-06	0.000109824	Up
MFSD14C	1.435289622	0.004276753	0.060277229	Up
MFSD9	5.260644559	1.53256E-07	1.74357E-05	Up
MGAM	1.407005626	2.64663E-06	0.000221763	Up
MGAM2	1.536511696	3.91313E-05	0.002057046	Up
MGAT1	1.10364254	0.031060468	0.213954341	Up
MIA3	1.133829953	0.01216867	0.119426033	Up
MIGA2	3.685434606	0.000204507	0.00712196	Up
MINDY4B	1.725839276	0.002057483	0.036824814	Up
MLKL	1.293666595	0.031442319	0.2156275	Up
MLX	2.281640754	0.027558072	0.200016428	Up
MME	1.044731081	8.01158E-05	0.003572244	Up
MMP9	2.353781393	3.3066E-06	0.00026504	Up
MOB1A	1.174751858	0.011216098	0.113317125	Up
MOSPD3	1.078373576	0.046042219	0.272745137	Up
MPG	2.856696642	0.009324749	0.100598896	Up
MSL3	1.387041193	0.048751884	0.281867262	Up
MSR1	1.25746211	7.39303E-07	7.35417E-05	Up
MSRB1	1.062246413	4.48037E-05	0.002299152	Up
MSRB2	1.363393856	2.03326E-13	3.18066E-11	Up
MT1E	2.120669806	7.61155E-05	0.003441609	Up
MTARC1	1.147953014	7.34076E-06	0.000528254	Up
MTDH	2.084528298	0.026210983	0.193842974	Up
MXD1	1.325623476	0.000217589	0.007443878	Up
MYADM	1.939751445	0.013411402	0.12686358	Up
MYBBP1A	1.790173579	0.038292437	0.243552765	Up
MYL9	1.797043055	4.89635E-05	0.002457359	Up
MYT1L	1.447811227	0.022753618	0.176510017	Up
MZB1	1.238871481	0.005452446	0.07053273	Up

NAMPT	1.181465261	7.40047E-05	0.003361842	Up
NAPB	1.933923268	0.036855882	0.238045755	Up
NAT1	1.006715986	0.0225365	0.175810082	Up
NAT8	2.456833036	0.001955459	0.035597846	Up
NCBP2	2.682068237	0.010524689	0.109172547	Up
NCK1	2.952947618	0.000826403	0.019765906	Up
NCOA1	2.13353428	0.010791432	0.110853173	Up
NCOA7	1.050061679	0.028221761	0.202677379	Up
NDST1	1.185470585	0.001254941	0.026288036	Up
NDUFAF3	1.191200542	0.008016872	0.091179879	Up
NECTIN2	1.309954021	0.003178315	0.049618797	Up
NEK2	1.987610226	0.001487296	0.029340273	Up
NHLH1	1.194521375	0.003577368	0.053543206	Up
NIP7	1.205205867	0.02493395	0.18739105	Up
NLRC4	1.229745548	2.61581E-09	3.45172E-07	Up
NLRX1	1.320498125	0.016196076	0.14294044	Up
NONO	1.925549438	0.038034835	0.242601055	Up
NOTCH2	1.182697533	0.000112063	0.004561899	Up
NSDHL	2.123512385	0.007183853	0.084399971	Up
NT5C3A	1.919450763	2.91272E-06	0.000238899	Up
NTNG2	1.194635846	0.000115587	0.004671056	Up
NUPR1	1.641124331	0.000452408	0.012518443	Up
OAS1	1.825250909	0.014872463	0.135186785	Up
OASL	1.073875642	0.009288831	0.100378883	Up
ODF3B	1.543760526	9.26341E-08	1.07597E-05	Up
OPLAH	1.527094158	0.000294631	0.009255246	Up
OR2B2	1.381500453	0.001685485	0.032116111	Up
OR2B6	1.63137765	0.000259359	0.008498159	Up
OR52B4	1.333111975	0.000210695	0.007252789	Up
ORM1	1.172658241	0.003238887	0.050315033	Up
ORMDL1	2.105131612	0.001330789	0.027419702	Up
OSCAR	1.417347497	0.015182247	0.137094552	Up
P2RY14	2.447608749	4.2227E-07	4.46375E-05	Up
PACSIN2	1.014653639	0.021468374	0.170538769	Up
PADI2	1.636919171	2.7328E-07	2.97807E-05	Up
PADI4	1.187779304	1.68512E-05	0.001031136	Up
PANK2	1.031477736	0.025678062	0.191499506	Up
PARG	1.369085943	0.019208296	0.159371615	Up
PARP14	1.103604213	0.000278374	0.008895806	Up
PARP9	1.114029723	5.77361E-06	0.000431575	Up
PCCB	3.07355463	0.011288125	0.113746184	Up
PCOLCE2	1.555646072	0.049261511	0.283476779	Up
PDCD1LG2	2.610466873	8.8167E-08	1.03337E-05	Up
PDLIM7	1.189185374	0.000833393	0.019896285	Up

PDZD4	2.457700667	0.009011577	0.098316841	Up
PEAK3	1.035942282	4.55954E-05	0.002330532	Up
PF4V1	1.290632837	0.020922974	0.168055236	Up
PFKFB3	1.051006951	0.000259631	0.0084999	Up
PFKFB4	1.133972297	0.012343314	0.120402762	Up
PGD	1.235972486	0.000219462	0.007488139	Up
PGLYRP1	1.395012495	3.30432E-06	0.00026504	Up
PHC2	1.233418185	1.36136E-07	1.5554E-05	Up
PHTF1	1.169887903	0.009591647	0.102622155	Up
PIAS1	1.073126648	0.028572453	0.204089716	Up
PICALM	1.039423705	0.002266503	0.039412361	Up
PLAC8	1.051074156	0.016514997	0.144954599	Up
PLAGL1	1.142523657	0.015022462	0.136104547	Up
PLAUR	1.250716974	1.09003E-06	0.000103653	Up
PLBD1	1.372014248	6.93246E-08	8.32646E-06	Up
PLCD4	1.064006388	0.00759072	0.087800235	Up
PLEKHA8	1.321262112	0.044974394	0.26896587	Up
PLSCR1	1.376957095	7.25097E-05	0.003310978	Up
PLSCR2	1.245389171	0.034307217	0.227240649	Up
PML	1.292742283	0.000846041	0.020111615	Up
PNISR	1.04884025	0.012537278	0.121444121	Up
PNPLA1	1.328256206	0.031918854	0.217664256	Up
POLR1B	2.22297661	0.003009952	0.047955266	Up
PPHLN1	2.077260858	0.000920331	0.021392599	Up
PPP4R3A	1.325647708	0.030707109	0.212274109	Up
PRCP	3.19816092	0.000186947	0.006678291	Up
PRELID1	1.049900644	0.032533152	0.220150639	Up
PRKAG1	1.095236006	0.044169166	0.266188155	Up
PRPF40A	1.410607588	0.022299565	0.174664166	Up
PRR7	1.279593755	0.001053566	0.023517781	Up
PRRG4	1.029082058	0.001418013	0.028474016	Up
PSMB9	1.078571281	1.04932E-07	1.21517E-05	Up
PSME3IP1	1.174458647	0.007130904	0.083984033	Up
PSRC1	1.549496688	0.025338606	0.189624078	Up
PSTPIP2	1.831105479	4.70565E-08	5.72275E-06	Up
PTGDR	1.585391293	0.009634011	0.103018596	Up
PTGR1	1.202198492	0.007347308	0.085725961	Up
PTPRC	1.014358869	0.0002055	0.007131136	Up
PXT1	2.485250877	2.60627E-05	0.001491303	Up
PXYLP1	1.504560758	0.034649852	0.228534683	Up
PYGL	1.083462751	6.2491E-06	0.00046266	Up
QDPR	3.178128372	0.000605109	0.015598132	Up
QPCT	1.034896838	0.000261256	0.008538694	Up
QSOX1	2.111734382	0.000137726	0.005353805	Up

RAB20	1.457757207	4.51192E-09	5.85418E-07	Up
RAB33B	1.035885875	2.07657E-05	0.001228059	Up
RAB43	1.700281335	0.007724896	0.088875249	Up
RAB5IF	1.095424295	0.003566677	0.053507051	Up
RALB	1.220593215	0.000102289	0.004280807	Up
RAP1B	3.536512773	3.18386E-05	0.001762024	Up
RARG	2.576108455	0.012275313	0.12006562	Up
RASA2	1.229460717	0.001430879	0.028599149	Up
RASGRP4	1.713255914	0.000994952	0.022612278	Up
RBBP4	1.790283398	0.007847041	0.089854174	Up
RBM23	1.162011508	0.012659976	0.122327216	Up
RBPJ	1.082726057	0.016921773	0.147358063	Up
RC3H1	1.523598693	0.004125982	0.05908729	Up
RCAN1	1.225898796	0.001444435	0.028766362	Up
RCN2	2.717055165	0.002728543	0.04483447	Up
RD3L	1.879271317	0.008660331	0.095774674	Up
RETREG1	1.377359988	0.008864235	0.097116069	Up
RGMA	1.472706612	0.00499205	0.06673555	Up
RGS16	1.567423178	0.000269463	0.008732292	Up
RGS3	1.050935849	2.90288E-06	0.000238899	Up
RLIM	1.155628163	0.025002675	0.187762052	Up
RNF146	1.505299248	0.007476819	0.086906127	Up
RNF24	1.493338399	0.000989022	0.022490675	Up
RO60	1.108060668	0.020086537	0.16353771	Up
ROPN1L	1.024626577	0.012438221	0.120876953	Up
RPS9	2.17066355	0.003325534	0.051074468	Up
RRAGB	1.550516648	0.023608356	0.180790793	Up
RRM2B	1.183698977	0.023529069	0.180447722	Up
RSAD2	1.49166132	0.032448322	0.219921844	Up
RSPH1	1.495052296	0.049037648	0.282910938	Up
RSRC1	1.549437794	0.035805304	0.233533548	Up
RTN3	1.070502071	0.000863103	0.020392252	Up
RTP4	1.540435517	0.000191663	0.006802909	Up
RUFY4	1.171229479	0.001552189	0.030214327	Up
RXRA	1.07501655	0.021563009	0.171070948	Up
S100A12	1.604030547	0.001423136	0.028488868	Up
S100A8	1.836650397	8.23837E-05	0.003652657	Up
S100A9	1.549355692	3.51455E-06	0.0002794	Up
SAMD9L	1.624073833	0.001348127	0.027643011	Up
SAP130	2.880388729	0.019011194	0.158506067	Up
SAT1	1.076118694	1.53698E-05	0.000955837	Up
SBNO2	1.167995471	0.000182138	0.006582308	Up
SCARF1	1.752731756	1.5669E-06	0.000138785	Up
SCO2	1.375722653	0.048874309	0.282280604	Up

SCRIB	1.100963345	0.030460199	0.211402967	Up
SDC3	1.866140394	1.34521E-06	0.000122219	Up
SDF4	1.104741516	0.014164124	0.13123888	Up
SECTM1	1.536734405	8.35305E-08	9.87978E-06	Up
SELL	1.04287955	1.49241E-05	0.000935346	Up
SEMA4B	1.078374197	0.015696012	0.140338048	Up
SEPTIN11	3.760859911	7.9291E-05	0.003547974	Up
SEPTIN4	1.636835005	0.00493568	0.066233039	Up
SERINC2	1.480083002	0.030937241	0.213256978	Up
SERPINA1	1.459290893	3.49702E-05	0.001897439	Up
SERPING1	2.844317629	7.28023E-08	8.6637E-06	Up
SFN	2.071790918	1.5593E-05	0.000967889	Up
SH2D3C	1.007244664	0.038773283	0.244985263	Up
SH3PXD2B	1.155772832	0.000539674	0.014299508	Up
SHISA5	1.008101929	0.026597679	0.195378233	Up
SIGLEC11	1.449218076	0.007858726	0.089961428	Up
SIPA1L1	1.387200343	5.24891E-11	7.35131E-09	Up
SIPA1L2	1.238024719	0.000385507	0.011211203	Up
SIRPB1	1.793931314	0.021177436	0.169266043	Up
SIRPB2	1.279411253	0.00016227	0.006041507	Up
SLA	1.245380918	0.00021611	0.007412897	Up
SLC16A3	2.346901502	0.001503501	0.029560516	Up
SLC19A1	1.061876703	0.001402945	0.028273905	Up
SLC25A39	4.421660855	1.49607E-05	0.000936128	Up
SLC26A8	2.935372742	0.0070354	0.083289084	Up
SLC2A14	1.195295517	0.037711764	0.241383911	Up
SLC2A3	1.104597382	0.000131773	0.005155683	Up
SLC35A5	1.769638719	0.042691947	0.26116877	Up
SLC36A1	1.998294431	7.37926E-05	0.003356138	Up
SLC38A2	1.154672818	0.021205342	0.169374208	Up
SLC38A6	1.506088454	0.009912035	0.105295015	Up
SLC43A2	1.938994656	0.01345888	0.12725743	Up
SLC44A1	2.691067569	0.015396775	0.138253045	Up
SLC6A12	2.137428865	0.003324193	0.051074468	Up
SLC7A11	1.047054655	0.00136875	0.027838627	Up
SLU7	2.375708523	7.75837E-05	0.003491716	Up
SMG6	3.105093776	0.000124173	0.004923939	Up
SMIM1	1.240388178	0.032526383	0.220150639	Up
SMOX	1.00007276	0.043301432	0.263506087	Up
SMPDL3A	1.087220275	0.000423847	0.012028634	Up
SMTNL1	1.767871127	3.23003E-06	0.000261061	Up
SNAPC3	1.74340162	0.015262884	0.137543222	Up
SNX10	1.168377655	0.000224421	0.007630516	Up
SOAT1	2.154597742	0.000680391	0.016992223	Up

SOCS1	2.080862908	9.17016E-07	8.87173E-05	Up
SOCS3	1.291311008	0.000205976	0.007141038	Up
SOD2	1.622055303	4.92893E-07	5.09914E-05	Up
SORT1	1.333346735	0.000104055	0.004331362	Up
SPAG9	1.83621214	1.24072E-05	0.000800895	Up
SPATC1	1.23775216	0.032564889	0.220211757	Up
SPDYC	1.912437506	0.007340821	0.085698702	Up
SPHK2	1.339396552	0.043796006	0.264878409	Up
SPIDR	1.084901224	0.000125491	0.004962696	Up
SQOR	1.094666114	2.71109E-07	2.96273E-05	Up
SRA1	1.343927646	0.000548203	0.014477561	Up
SRGN	1.27242031	2.58575E-06	0.000217601	Up
SRP68	1.573430192	0.011233355	0.113400475	Up
SRPK1	1.022000704	9.86766E-06	0.000669258	Up
SRPRA	1.043174738	0.017579486	0.150750695	Up
STARD3NL	1.372163803	0.00802336	0.091200186	Up
STAT1	1.156120103	1.05672E-06	0.000100974	Up
STEAP4	1.179945485	0.017714978	0.151596528	Up
STRADA	1.046675077	0.000619396	0.015819276	Up
STUB1	1.79806254	0.001565083	0.030389085	Up
STX11	1.214431734	2.35563E-07	2.61105E-05	Up
SUCNR1	1.055805854	0.002052882	0.036794193	Up
SUPT6H	1.648143005	0.025715186	0.19170266	Up
SVIL	1.004005164	0.00622276	0.076444572	Up
SYK	1.313340429	0.001805591	0.033612243	Up
SYN2	1.825875447	0.002730198	0.04483447	Up
SYNJ1	1.462576397	0.038918752	0.245739181	Up
SYNRG	2.363651207	0.000931059	0.02154162	Up
TAGLN2	1.922969363	0.009545199	0.102215224	Up
TANGO2	1.03285486	0.003485535	0.052820833	Up
TAP1	1.144806121	5.16832E-06	0.000395106	Up
TAPBP	1.66926332	0.013626417	0.128056596	Up
TAS2R40	1.214513986	0.001185162	0.02537437	Up
TBC1D10C	1.346010919	4.61849E-05	0.002351369	Up
TBC1D14	1.092304305	0.009718792	0.103715628	Up
TBC1D5	1.611642716	0.007691018	0.088645987	Up
TBC1D8	1.822536554	0.034045296	0.22627844	Up
TBL1X	2.808154821	4.70892E-05	0.002381781	Up
TBL1XR1	1.023688171	0.030689721	0.212229543	Up
TCIRG1	1.071956181	5.62145E-06	0.000421826	Up
TDRD9	1.60896948	1.75584E-05	0.001066008	Up
TFEB	1.12161091	0.028395976	0.203288776	Up
TFEC	1.059761018	0.021317256	0.169893445	Up
THADA	1.141705415	0.048936569	0.282482353	Up

TIFA	1.080337651	0.000617102	0.01579188	Up
TIMM10	1.059880665	0.001750447	0.032955408	Up
TLK2	1.027494481	0.043542974	0.264226818	Up
TLR2	1.095875251	0.000905239	0.021092344	Up
TLR4	1.202579566	2.66967E-07	2.9257E-05	Up
TLR5	1.521213284	4.6812E-06	0.000362489	Up
TM9SF1	1.47218371	0.04710385	0.276390923	Up
TMBIM4	1.54739717	0.0015483	0.030153767	Up
TMCO6	1.529163417	0.003143276	0.049349817	Up
TMEM140	1.15345915	3.65992E-06	0.000289178	Up
TMEM158	1.687279257	0.005180031	0.068205882	Up
TMEM164	1.106316392	0.002061442	0.036871204	Up
TMEM183A	1.046210738	0.014821662	0.134951992	Up
TMEM272	1.135122617	0.017512005	0.150371459	Up
TMEM30A	1.084122964	0.012330406	0.120371947	Up
TMEM39B	1.5852171	0.018834801	0.157545517	Up
TNFAIP2	1.409788944	0.000475397	0.012969786	Up
TNFAIP6	2.277141408	4.62017E-07	4.83126E-05	Up
TNFSF10	1.105116618	0.000113993	0.004621045	Up
TNFSF13B	1.095973781	1.16137E-05	0.00075851	Up
TNK2	1.238252346	0.036016391	0.234556974	Up
TOPORS	3.029955878	0.001307817	0.02707405	Up
TP53	2.787677009	0.000192395	0.006822627	Up
TP53I11	1.977514153	0.000111941	0.004561714	Up
TPD52L2	1.099624285	0.029519882	0.207543278	Up
TPST1	1.813644261	2.94916E-05	0.001655755	Up
TPX2	1.023366387	0.008299191	0.093221502	Up
TREML4	1.242984928	0.000205505	0.007131136	Up
TRIB1	2.341905008	0.006357789	0.077465584	Up
TRIM22	1.326506437	1.01999E-05	0.000688629	Up
TRMO	1.508678436	0.00229806	0.03976504	Up
TRMT1	2.119391676	0.018423625	0.155143159	Up
TRPV4	1.264191016	0.032985261	0.221509988	Up
TSHZ3	1.106801864	3.58539E-07	3.86376E-05	Up
TSPO	1.24139517	0.031353722	0.215286308	Up
TTC26	1.703349232	0.047824465	0.278906151	Up
TTC7A	1.631708301	0.016961168	0.147662983	Up
TTI2	1.024198818	0.000172506	0.006337471	Up
TYMP	1.398026232	1.29813E-06	0.000119057	Up
UBAP1	1.717361965	0.00015164	0.005750616	Up
UBE2L6	1.547750512	8.91779E-08	1.04207E-05	Up
UBE4B	1.124642855	0.006487593	0.078529229	Up
UNC119	2.641327318	0.000274283	0.008823236	Up
USP1	2.32331977	0.002885987	0.046592544	Up

USP15	1.330405285	7.23382E-05	0.003310978	Up
VAMP5	1.235619863	1.1219E-05	0.000741464	Up
VEZT	2.869708919	8.66158E-05	0.003811309	Up
VMP1	1.156046392	1.8024E-08	2.24836E-06	Up
VNN1	2.429228427	1.21731E-08	1.54332E-06	Up
VPS37A	2.918693104	4.44651E-05	0.002284801	Up
VPS50	1.2690983	0.006698883	0.080328759	Up
VPS9D1	1.297752152	7.71336E-10	1.03903E-07	Up
WARS1	2.071426137	1.90185E-07	2.13861E-05	Up
WDR1	1.156157975	0.031701788	0.21672803	Up
WIPI2	1.512195742	0.008090165	0.091690899	Up
XAF1	1.169686818	0.004828699	0.065265366	Up
XRN1	1.05645753	0.000108157	0.004454314	Up
YAF2	1.37795423	0.010432244	0.108718643	Up
ZBP1	1.033933026	0.010845811	0.111224757	Up
ZC3HAV1	1.262890678	0.001536118	0.029976704	Up
ZCCHC7	2.322621975	0.022363773	0.174917875	Up
ZCWPW1	1.068630333	3.60177E-05	0.00193533	Up
ZDHHC12	1.059485893	6.66838E-06	0.000488113	Up
ZDHHC17	1.082278393	0.003104546	0.048840578	Up
ZFYVE27	1.072650209	0.000159522	0.00596556	Up
ZMAT5	1.655246078	0.025804165	0.192181339	Up
ZNF106	1.354575411	0.001076545	0.023851833	Up
ZNF155	2.017235596	0.008381282	0.093900204	Up
ZNF185	1.022778236	0.003279851	0.050653592	Up
ZNF189	1.180804703	0.017722808	0.151611097	Up
ZNF254	1.260009537	0.04943342	0.283987788	Up
ZNF26	1.77206392	0.025677093	0.191499506	Up
ZNF267	1.068191164	1.03478E-05	0.000694535	Up
ZNF33B	3.567809682	0.00022577	0.007669661	Up
ZNF438	1.041266288	0.00012954	0.005086847	Up
ZNF518A	1.553119698	0.018248908	0.154173861	Up
ZNF565	2.095522737	0.000211687	0.007280496	Up
ZNF611	1.197047808	0.029845595	0.208773865	Up
ZNF655	1.423336363	0.029904312	0.208958536	Up
ZNF671	1.622390646	0.045088887	0.269484419	Up
ZNF808	1.119265639	0.022688969	0.176276195	Up
AAGAB	-2.50468508	0.029860333	0.208808014	Down
ABCB4	-1.029226245	0.045198621	0.269849263	Down
ABR	-2.00939205	0.022364411	0.174917875	Down
ABRAXAS1	-1.632085729	0.017630653	0.151089284	Down
ACACA	-2.711056246	1.19731E-05	0.000778052	Down
ACE	-1.125240333	0.030782129	0.212565447	Down
ACOT11	-1.094689018	0.006927406	0.08233723	Down

ACSM1	-1.193194004	0.003074799	0.048589347	Down
ACTA2	-1.724722608	0.036284346	0.235826973	Down
ADAM23	-1.342470916	0.002065227	0.036904879	Down
ADAMDEC1	-1.075366077	0.014278014	0.131931635	Down
ADAMTS10	-1.068181638	0.006613113	0.079644559	Down
ADD1	-1.822895509	0.010741148	0.11068776	Down
ADGRB2	-1.138502751	0.038178833	0.243240723	Down
ADGRG6	-1.229835562	0.012508904	0.12132073	Down
ADH6	-1.298350091	0.037954642	0.242338766	Down
ADORA3	-2.251437898	0.030436818	0.211381925	Down
AEBP2	-1.741172999	0.000931184	0.02154162	Down
AFDN	-1.091599047	0.022728899	0.176397119	Down
AFF3	-3.11672016	0.000510971	0.013737435	Down
AGAP1	-1.011234626	0.014561067	0.133419126	Down
AGL	-1.324517827	0.002956062	0.04732994	Down
AGPAT1	-2.019904382	0.005097601	0.067680157	Down
AHNAK	-1.001683263	0.0010809	0.023920993	Down
AJAP1	-1.344262591	0.004543114	0.062789495	Down
AK3	-1.034633391	0.004672246	0.064004163	Down
AKR1C4	-1.169086439	0.007890153	0.090241295	Down
AKT3	-1.120225096	0.003718561	0.05520917	Down
ALOX15	-1.321541168	0.016842227	0.146830157	Down
AMH	-1.714400958	1.66093E-05	0.001021169	Down
AMZ2	-1.226379354	0.002498965	0.042224451	Down
ANAPC11	-1.321530615	0.001857395	0.034395054	Down
ANK3	-1.213846335	0.007355162	0.085791798	Down
ANKRD36B	-1.055509399	0.001195832	0.025504296	Down
ANKRD46	-1.135232686	0.00996493	0.105683291	Down
ANXA4	-1.538172725	0.010985438	0.112159511	Down
AP1G2	-1.084209106	0.002175432	0.038274774	Down
AP2M1	-1.329618965	0.013246983	0.125756334	Down
AP3S1	-2.412885971	0.000405652	0.01164862	Down
APBA2	-2.031219078	0.014605048	0.133601238	Down
ARHGAP42	-1.12689613	0.000381414	0.011132766	Down
ARHGEF25	-1.453540743	0.04937606	0.283742294	Down
ARHGEF28	-1.262461963	0.018494721	0.155539282	Down
ARHGEF9	-1.452855204	0.026236615	0.193919505	Down
ARIH2	-1.42517034	0.000256054	0.008446958	Down
ARL4A	-1.786175821	0.011404899	0.114684562	Down
ARMC5	-2.040660358	0.010199313	0.107172902	Down
ASH1L	-1.692813814	0.037047791	0.238788676	Down
ASTN2	-1.416798805	0.001247427	0.026187191	Down
ATP13A4	-1.036329337	0.002366543	0.040641895	Down
ATP1A1	-1.791988158	0.002246322	0.03911404	Down

ATP5MD	-1.012892133	0.039920589	0.249672619	Down
AURKC	-1.316169404	0.013580516	0.12789596	Down
AUTS2	-1.274685132	0.000381949	0.011132766	Down
B3GALNT2	-1.706097327	0.023343135	0.179397173	Down
B9D1	-1.488529146	0.004673994	0.064005505	Down
BACH2	-1.575110091	1.33539E-05	0.000854217	Down
BANK1	-1.51517838	0.035568765	0.232461293	Down
BCL7A	-1.566383982	0.029203213	0.206138764	Down
BECN1	-1.302088637	0.028158013	0.202479169	Down
BICD1	-3.282912741	6.08191E-07	6.17664E-05	Down
BLNK	-1.177176431	0.008145315	0.092086761	Down
BNC2	-1.071061967	0.006010914	0.07482881	Down
BRD2	-1.004717138	0.0230654	0.178021474	Down
BTBD1	-1.262171658	0.0442554	0.266514782	Down
BTNL9	-1.235498287	0.02699812	0.197026347	Down
BUD23	-1.711201537	0.001566268	0.030396874	Down
C12orf71	-1.356362175	0.038511589	0.244165436	Down
C19orf12	-1.267353636	0.012432932	0.120855825	Down
C19orf48	-1.052810524	0.047222996	0.276769633	Down
C20orf173	-1.219497304	0.021958294	0.173039207	Down
C21orf59-TCP10L	-1.192104778	0.040386607	0.251654098	Down
C2CD2	-1.191321822	0.042095744	0.258782202	Down
C5orf63	-1.173563195	0.000162253	0.006041507	Down
C6orf201	-1.044355677	0.03842887	0.243921468	Down
CAMK2D	-1.187580231	0.047249524	0.276769633	Down
CAMK2N1	-1.618909264	0.000196315	0.006906523	Down
CAMKV	-1.241400869	0.008599207	0.095507077	Down
CAND1	-2.704454954	0.006392987	0.077706566	Down
CAPN14	-1.511389729	0.000117994	0.004740861	Down
CARD11	-1.061771577	0.00618351	0.076082867	Down
CARNMT1	-1.963753601	0.010874528	0.111364857	Down
CAVIN4	-1.066467848	0.02678341	0.196271702	Down
CBFA2T2	-1.763551278	0.019291852	0.159613434	Down
CBLB	-1.544304484	0.000181292	0.006573107	Down
CBLN3	-1.35611631	0.002747544	0.045032095	Down
CCDC113	-1.251410212	0.049041785	0.282910938	Down
CCDC188	-1.218245412	0.014664512	0.13405036	Down
CCR6	-1.53614263	3.13843E-07	3.41052E-05	Down
CD79B	-1.153995665	0.032805815	0.220877022	Down
CDC25B	-1.183871674	0.021895153	0.172646843	Down
CDHR3	-1.256167351	0.000636799	0.016132802	Down
CDK10	-1.839092218	0.049660578	0.28470544	Down
CEP78	-1.099404174	0.027755013	0.200785605	Down
CEP83	-1.205034223	0.04680225	0.27506337	Down

CGN	-1.642157166	0.011169125	0.113046232	Down
CISH	-1.378684453	0.015936401	0.141508967	Down
CLDN24	-1.346652212	0.012356641	0.120476471	Down
CLNK	-1.283117958	0.001913659	0.035124074	Down
CMTM3	-1.829873809	0.023151052	0.178486697	Down
COL11A2	-1.030714395	0.019281706	0.159597568	Down
COL19A1	-1.066080104	0.019355824	0.159903998	Down
COL4A3	-1.314390015	3.54376E-05	0.001917439	Down
COL5A3	-1.644442729	0.003858225	0.056440355	Down
COL9A1	-1.591758951	0.012094441	0.118996661	Down
COLQ	-2.551857339	3.55803E-05	0.001919802	Down
COMMD3-BMI1	-1.172288209	0.038657987	0.244657711	Down
CORO1C	-2.036243267	0.040644995	0.252675954	Down
COX18	-1.55996248	0.005352594	0.069682505	Down
CRLS1	-1.426530391	0.014972315	0.13583979	Down
CRTAC1	-1.221865173	0.001137463	0.024680013	Down
CSMD1	-3.282738927	2.52564E-05	0.001449443	Down
CSPP1	-1.189624917	0.034848595	0.229183121	Down
CSRP1	-3.015049172	0.004192361	0.059554246	Down
CSTF1	-1.660611016	0.007472322	0.086897101	Down
CTH	-1.134305504	0.033385417	0.223382468	Down
CTLA4	-1.116053704	0.033956203	0.225841057	Down
CXXC4	-1.010354843	0.028388584	0.203288776	Down
CYFIP2	-4.916819481	8.58367E-08	1.01217E-05	Down
CYP20A1	-1.116201716	0.030544556	0.211670436	Down
CYP27B1	-1.305655492	0.015392064	0.138253045	Down
DAB1	-1.570540407	0.00289904	0.046725484	Down
DACT3	-1.231331871	0.003572861	0.05352632	Down
DCAF1	-1.029238074	0.048760021	0.281872301	Down
DCBLD1	-1.265224117	0.012776396	0.12297964	Down
DCHS2	-1.167130295	0.011230168	0.113397802	Down
DEF8	-2.490583219	3.83151E-05	0.002020829	Down
DELE1	-1.64057158	0.006527558	0.078865347	Down
DEPDC5	-1.034255206	7.99876E-06	0.000561938	Down
DGKK	-1.428635038	0.004877654	0.065704377	Down
DHX8	-1.408445242	0.04272502	0.261314386	Down
DIP2A	-1.007930676	0.005948151	0.074342306	Down
DIRAS1	-1.001504744	0.041269956	0.255353738	Down
DLAT	-3.455822444	0.00017324	0.006346422	Down
DLG2	-1.198408334	0.009532146	0.102182816	Down
DNAAF1	-1.226612978	0.041701066	0.257241671	Down
DNAJC21	-1.161885926	0.037216327	0.23935799	Down
DNMBP	-1.269406841	0.00190914	0.035092663	Down
DOCK4	-5.061320779	7.1975E-08	8.61812E-06	Down

DOCK7	-1.84963143	0.002050056	0.036769263	Down
DPH2	-1.643026027	0.049207347	0.283429101	Down
DSE	-1.864950684	0.026475968	0.194881971	Down
DTHD1	-1.467921563	0.030670774	0.212192136	Down
DTYMK	-1.452138525	0.012262239	NA	Down
DYRK2	-1.075717644	0.00100862	0.022848707	Down
EBF4	-1.013858052	0.014349336	0.132354375	Down
EDIL3	-1.44957052	0.047235176	0.276769633	Down
EFNA5	-1.906548497	0.000461427	0.012686776	Down
EHBP1	-2.280538418	0.005314204	0.069387433	Down
EHMT1	-1.993074746	0.000392303	0.011366238	Down
EHMT2	-1.028621045	5.46547E-05	0.002667081	Down
EIF2AK3	-1.868799209	0.001638587	0.031469794	Down
EIF2S3B	-1.1177496	0.047893728	0.279110284	Down
EIF4E	-1.174908533	0.02862753	0.204155335	Down
ELOVL4	-1.419660652	0.004737782	0.064469392	Down
ENAM	-1.077283753	0.031976938	0.217956883	Down
ENTPD6	-1.85643342	0.020976005	0.168293254	Down
EPN3	-1.046711889	0.045785438	0.271964265	Down
ERAP2	-2.374699677	0.019108682	0.158843224	Down
ERICH1	-1.212726652	0.001320008	0.027239199	Down
ERVW-1	-1.059477	0.010782597	0.110851103	Down
EXTL1	-1.203144468	0.019950231	0.162906588	Down
FAM110C	-1.452910952	0.002670392	0.044140549	Down
FAM156B	-1.965693216	0.003036243	0.048196014	Down
FAM171A2	-1.562893625	0.001427683	0.028549983	Down
FAM193A	-2.024691551	0.026025663	0.193054961	Down
FAM221B	-1.169066531	0.020431947	0.165447172	Down
FAM241B	-1.179737491	0.000713603	0.017622012	Down
FAM3C	-2.370948514	0.003997509	0.057953425	Down
FAM81A	-2.053974794	0.000158566	0.005947875	Down
FASTKD2	-1.513720813	0.045282059	0.270222655	Down
FAT4	-1.065462277	0.0132283	0.125665125	Down
FCRL1	-1.070765993	0.003676957	0.054696142	Down
FCRL2	-1.583539527	0.048938366	0.282482353	Down
FCRL5	-1.230926189	0.041595309	0.256882115	Down
FCRLA	-1.499774265	0.029091871	0.205807721	Down
FDFT1	-1.040221929	0.005237137	0.068775872	Down
FEZ1	-1.411287757	0.003539184	0.053327356	Down
FGF9	-1.221795192	0.033002339	0.221586315	Down
FHAD1	-1.117258814	0.004929729	0.066176071	Down
FHOD1	-3.348789293	1.20008E-06	0.000112186	Down
FILIP1L	-1.912493577	0.000885118	0.020777251	Down
FKTN	-2.52175618	0.001060905	0.023626755	Down

FMN1	-1.39914533	0.011148418	0.112895558	Down
FOPNL	-2.068170379	0.019912993	0.162773821	Down
FOSB	-1.219648888	0.006063693	0.075108872	Down
FOXP1	-2.482678899	2.13111E-06	0.000184959	Down
FRS2	-1.755760202	0.017325685	0.149399853	Down
FSCN2	-1.134239025	0.000463852	0.012717421	Down
FTO	-3.521790439	6.70727E-09	8.6448E-07	Down
FZD4	-1.039167687	0.002040713	0.036618627	Down
G3BP1	-1.050934556	0.005328261	0.069458972	Down
GABPB2	-1.006503553	0.000977506	0.022320397	Down
GALNT11	-1.45234277	0.015795412	0.140934455	Down
GANAB	-2.237253546	0.003775051	0.05574919	Down
GAR1	-1.232792869	0.011100024	0.112493581	Down
GATM	-1.241852582	0.024624698	0.185858983	Down
GFOD1	-1.320325639	0.004556218	0.062858634	Down
GIT1	-2.892725502	0.001089331	0.024066402	Down
GJA3	-1.599466119	0.049157515	0.283283687	Down
GLI3	-1.413826851	0.009698467	0.103565105	Down
GLOD4	-1.316114777	0.041258366	0.255322746	Down
GLOD5	-1.253019097	0.015190699	0.137115677	Down
GLRA3	-2.165051006	0.000310792	0.009546473	Down
GLYATL1B	-1.519205862	0.000924261	0.0214344	Down
GNA12	-1.686863649	0.001381277	0.027980963	Down
GNLY	-1.163874738	0.038492092	0.244123054	Down
GOSR2	-2.459963685	0.010292703	0.107651094	Down
GPATCH8	-1.101639157	0.000663269	0.01669694	Down
GPN1	-1.089384298	0.017122385	0.148360466	Down
GPSM2	-1.242400225	0.013101268	0.124972636	Down
GRHL2	-1.519065609	0.000312522	0.009561753	Down
GRIK4	-1.628103839	0.044490571	0.267459156	Down
GRM3	-1.015500086	0.007646374	0.088286031	Down
GSTA4	-1.141527012	0.026546021	0.195121804	Down
GTDC1	-1.486049095	0.014467501	0.133064651	Down
GVQW3	-1.026245709	0.046969238	0.275794096	Down
GYPB	-3.269119779	7.5329E-06	0.000537204	Down
HDAC9	-1.993143292	0.007587317	0.087787042	Down
HERC4	-1.146694797	0.007839128	0.089790068	Down
HES1	-1.092015012	0.011084829	0.112427695	Down
HEXA	-1.506936284	0.019595728	0.161219953	Down
HIPK1	-2.057735034	0.013865383	0.129429149	Down
HLF	-1.130006671	0.023988362	0.182710953	Down
HMGA1	-1.292137497	0.002839538	0.046053457	Down
HMOX2	-1.431364369	0.008052326	0.091422298	Down
HOXC4	-1.160042852	0.018035062	0.153368668	Down

HS3ST1	-1.012972619	0.001890179	0.034831222	Down
HTD2	-1.94128571	0.029720037	0.208346376	Down
HYDIN	-2.068725325	9.4652E-05	0.004043682	Down
ICAM2	-1.249164929	0.010759007	0.11080321	Down
IDE	-2.256897048	0.008560755	0.095298278	Down
IFNG	-1.223613536	5.24122E-05	0.002590232	Down
IGSF8	-2.222345423	0.005115204	0.067772604	Down
IL23R	-1.710902223	0.000628932	0.016031163	Down
IL5	-1.481912214	0.038194848	0.243240723	Down
IMMP2L	-1.163509919	0.010032432	0.106163266	Down
INO80E	-1.372914901	0.032800719	0.220877022	Down
INPP4A	-1.966552482	0.004700223	0.06423725	Down
INVS	-1.30380135	0.01428318	0.131932374	Down
IPCEF1	-1.856293942	0.00397092	0.057675718	Down
IQGAP2	-4.570831706	1.00375E-08	1.27673E-06	Down
IRF6	-2.066747741	0.001838444	0.034141899	Down
ISM1	-1.224624999	0.002501801	0.042253969	Down
ITGB1	-4.010738718	1.75605E-07	1.98041E-05	Down
ITGB1BP1	-2.189714973	0.027410728	0.19921304	Down
JUP	-1.83990014	0.029241145	0.206368971	Down
KCNA6	-1.089422616	0.00550734	0.070935344	Down
KCNC4	-1.080860159	0.021970555	0.173067149	Down
KCND3	-1.118069745	0.005000147	0.066751797	Down
KCNH1	-1.037681616	0.000311404	0.009547993	Down
KCNH8	-1.436089951	0.002717196	0.044723643	Down
KCNJ11	-1.305752199	0.022555922	0.175861831	Down
KCNJ6	-1.380944535	0.02856171	0.204089716	Down
KDM4E	-1.118624862	0.015492441	0.138966299	Down
KHDRBS2	-1.375089981	0.021909153	0.172722125	Down
KIF5C	-1.070350521	0.001147528	0.024827103	Down
KIR2DL1	-1.65774411	0.036615092	0.237250471	Down
KLF8	-1.076905154	0.002080109	0.037085404	Down
KLK1	-1.482933469	0.013573823	0.12787692	Down
KPNA5	-2.521234244	0.003943993	0.057392046	Down
KRBA2	-1.372075528	0.004737386	0.064469392	Down
KRTAP5-2	-1.603195147	0.000143353	0.005500884	Down
KSR2	-1.612733679	0.00099763	0.02265987	Down
L1CAM	-1.084199851	0.047728265	0.27867892	Down
LAMA2	-1.330648879	1.39024E-06	0.00012565	Down
LAMA5	-1.117904834	0.013628795	0.128056596	Down
LARP7	-1.473137054	0.00531323	0.069387433	Down
LHB	-1.94572271	0.000350416	0.010441629	Down
LIMA1	-1.099156402	0.00548563	0.070796741	Down
LIMS1	-1.69386554	0.020491387	0.165824644	Down

LIX1	-1.538192466	0.00072713	0.017842522	Down
LNX1	-1.067424308	0.000691235	0.017168035	Down
LOC100132202	-1.052749735	0.033325612	0.223100449	Down
LOC100289561	-1.014038007	0.034705309	0.228783763	Down
LPIN1	-1.561895327	0.012311873	0.120281818	Down
LRFN2	-1.036686238	0.005616221	0.071931757	Down
LRIG2	-1.798398775	0.013917756	0.129730744	Down
LRMP	-5.744219675	4.12032E-11	5.79159E-09	Down
LRP1B	-1.372205447	0.000242284	0.008095963	Down
LRRC1	-1.127885998	3.81386E-05	0.002020829	Down
LRRC28	-1.918617368	0.018232024	0.154126037	Down
LRRN3	-1.824907534	0.000250675	0.00830481	Down
MAGED1	-1.130271124	0.022017909	0.173262633	Down
MAGI1	-1.009689489	0.045077485	0.269457784	Down
MAMSTR	-1.485634312	0.000500053	0.013481286	Down
MAN2C1	-1.134763722	0.005664023	0.072308033	Down
MAP3K7CL	-1.721620472	0.001727152	0.032637538	Down
MAPKAP1	-1.961494058	0.000690445	0.017159382	Down
MAPKAPK5	-1.016778798	0.005340742	0.06957491	Down
MAST4	-1.046145875	0.005634107	0.072023936	Down
MBNL2	-1.531699092	0.002659052	0.044045765	Down
MCM8	-1.671912095	0.022457156	0.175402731	Down
MCU	-3.359321478	1.85902E-09	2.46145E-07	Down
MEAF6	-4.467249449	4.57805E-07	4.81316E-05	Down
MECP2	-1.266065914	0.011284809	0.113742315	Down
METTL27	-1.561019669	0.002901602	0.046727959	Down
MEX3A	-1.083229775	0.022582221	0.175919132	Down
MICAL3	-1.021842636	0.000452182	0.012518443	Down
MIER3	-3.58834851	6.28517E-07	6.3664E-05	Down
MINK1	-2.061868787	0.008381915	0.093900204	Down
MKRN3	-1.748906695	0.000376004	0.010992511	Down
MLH1	-1.48004297	0.038405307	0.243891616	Down
MNS1	-1.428378991	0.010885982	0.111371744	Down
MPI	-2.36401597	0.046532041	0.274181428	Down
MRI1	-1.624199907	0.02649885	0.194959773	Down
MROH7	-1.278779665	0.00189621	0.034913838	Down
MRPL48	-3.467974538	4.78238E-05	0.002409513	Down
MRPS22	-1.402711721	0.010742091	0.11068776	Down
MRPS27	-2.013557958	0.007087773	0.083645131	Down
MS4A1	-1.424017446	0.003121048	0.049040527	Down
MSH2	-2.341708187	0.03725649	0.239576584	Down
MSTO1	-1.452841718	0.008454089	0.094463246	Down
MTFR1L	-1.397590434	0.006247191	0.076599166	Down
MTIF2	-1.215643021	0.034766205	0.228990651	Down

MUC20	-2.302233741	0.010873229	0.111364857	Down
MYBPC2	-1.037640008	0.029337901	0.206826071	Down
MYEF2	-2.177502706	0.012232001	0.119803202	Down
N4BP2	-1.212133081	0.011685996	0.1164493	Down
NAA38	-1.189907038	0.000487427	0.013223598	Down
NAF1	-1.077121648	0.001754278	0.033005435	Down
NAT9	-1.517470668	0.00273145	0.04483447	Down
NCAM1	-1.005743496	0.043087666	0.262711976	Down
NEDD1	-3.704005723	6.54998E-05	0.003087564	Down
NEK1	-2.914687093	0.006353254	0.077458986	Down
NELL2	-1.103542424	0.000921364	0.021403785	Down
NFKBID	-4.904826348	7.51526E-10	1.01587E-07	Down
NIBAN3	-1.927051118	0.00212511	0.037615968	Down
NINL	-1.10403974	0.024875165	0.187094231	Down
NIPAL3	-1.573102385	0.000610245	0.015680299	Down
NKAPD1	-1.522188254	0.023445153	0.180002912	Down
NMD3	-1.020304038	0.001131585	0.0246224	Down
NOLC1	-1.030149416	0.000368754	0.010842313	Down
NPRL3	-1.381583426	0.004682036	0.064070401	Down
NPTXR	-1.162313586	0.000178064	0.006474232	Down
NRL	-1.327868722	0.02910857	0.205807721	Down
NRXN2	-1.503173089	0.003056197	0.048374196	Down
NTN4	-1.21433319	0.013020748	0.124541395	Down
NUDT7	-1.607351	0.0137011	0.128482998	Down
NUP205	-2.516443146	0.017035726	0.148018135	Down
NUP93	-3.201175198	1.98507E-07	2.21934E-05	Down
NXPE1	-1.167297858	0.047147676	0.276548852	Down
OR2T11	-1.755080093	0.002508987	0.04233847	Down
OSBPL10	-1.215084222	0.00078253	0.018987642	Down
OSBPL3	-1.198629056	0.009545708	0.102215224	Down
PAM	-3.149725215	0.000469071	0.012806199	Down
PAQR3	-2.133614646	0.000535871	0.014235339	Down
PARP15	-1.347974013	0.015111587	0.136592268	Down
PARVA	-1.067633469	7.25092E-05	0.003310978	Down
PARVB	-1.389403958	0.003159867	0.049451929	Down
PATL2	-1.030550728	8.40816E-05	0.003715202	Down
PAWR	-2.119603816	0.000309392	0.009530792	Down
PAX5	-1.113848391	0.026133361	0.193492024	Down
PCCA	-1.217255275	0.008837552	0.096933229	Down
PCDHGA11	-1.150128453	0.000308198	0.009527126	Down
PCDHGA4	-1.209605622	0.033606552	0.224400378	Down
PCDHGA8	-1.189708361	0.002257519	0.039273745	Down
PCDHGA9	-1.122979463	0.002213004	0.038637941	Down
PCDHGB6	-1.161203069	0.003027451	0.048154962	Down

PCDHGC3	-1.546875783	1.02734E-05	0.000691083	Down
PCYT2	-1.887130807	0.005742659	0.07272815	Down
PDCD2L	-1.324056848	0.034248944	0.227048492	Down
PDE8A	-2.3225433	0.001697366	0.032248218	Down
PER3	-1.458132677	0.019480411	0.160659557	Down
PGA3	-1.033529325	0.017925206	0.152669231	Down
PGAM1	-1.01844873	0.022607397	0.175975747	Down
PGC	-1.5154044	0.004908412	0.066026989	Down
PHOSPHO2	-2.772990383	1.33507E-06	0.000121583	Down
PIGR	-1.177601237	0.030531045	0.211670436	Down
PIK3C2B	-1.183685673	0.00058729	0.015229897	Down
PLAAT5	-1.512431528	0.01361912	0.128056596	Down
PLEKHA1	-1.114033272	0.03852395	0.244165436	Down
PLEKHH2	-1.716753398	0.000837943	0.019980319	Down
PLVAP	-1.251880568	0.002170059	0.03821491	Down
PNPLA8	-1.9285508	0.006673293	0.08011017	Down
POGK	-1.016333123	0.011002674	0.11218064	Down
PPFIA3	-1.257131118	0.000342297	0.010262288	Down
PPP1R13L	-1.073070208	0.003265986	0.050519554	Down
PPP1R35	-1.062567146	0.031436622	0.215626547	Down
PPP2R5C	-2.207232488	3.16625E-05	0.001757293	Down
PPWD1	-2.015106188	0.00509572	0.067680157	Down
PRELID3B	-1.047572995	0.003877847	0.056663311	Down
PROM1	-1.910881565	0.027840166	0.201203289	Down
PRPF4	-3.865386451	4.76126E-05	0.002405118	Down
PRR4	-1.151597325	0.049683272	0.28470544	Down
PRSS1	-1.398102114	2.75511E-05	0.001562638	Down
PSMA3	-1.420366773	0.003174899	0.049605395	Down
PSPC1	-1.400010113	0.002091399	0.037218266	Down
PTCH1	-2.408771173	0.002755399	0.045141774	Down
PTGDR2	-1.006813313	0.027282924	0.198455226	Down
PTGDS	-1.057703801	0.010771363	0.110851103	Down
PTGES3	-1.51642113	0.001491725	0.0293913	Down
PTPRB	-1.097623226	0.001734761	0.032733487	Down
PWP1	-1.947338803	0.011921622	0.117834221	Down
PYROXD1	-1.115032878	0.004906958	0.066026989	Down
RAB11FIP3	-2.023148893	0.000205506	0.007131136	Down
RABEP1	-2.146897827	0.000100343	0.004231314	Down
RAD17	-1.651543788	0.011652295	0.11632805	Down
RAD51B	-3.077644152	0.000262439	0.008562936	Down
RAD51D	-1.618340569	0.033914113	0.225730347	Down
RAPGEF5	-1.076410103	0.000639187	0.016165091	Down
RAPGEF6	-1.029001965	0.007827832	0.089740171	Down
RARS2	-1.8699953	0.006939431	0.082454889	Down

RASA3	-1.176857299	2.25425E-05	0.001317075	Down
RASSF6	-1.107317796	0.030720543	0.212291318	Down
RBM28	-3.989212848	4.06544E-07	4.34077E-05	Down
RBPMS	-1.656629795	0.005401522	0.070102161	Down
RCC1L	-2.580560881	0.001842632	0.034186946	Down
RERE	-2.569709838	4.34817E-05	0.002249164	Down
REV3L	-1.637984386	0.029837193	0.208773865	Down
RFX3	-1.959378217	0.015758659	0.140703605	Down
RGS10	-6.184376948	4.04402E-10	5.54373E-08	Down
RHBDD2	-1.332923435	0.002683974	0.044327269	Down
RHOC	-2.367854209	0.001347572	0.027643011	Down
RNF14	-1.053341741	0.031805078	0.217155581	Down
RNF144A	-1.667207725	0.019214511	0.159381429	Down
RNF145	-1.370833477	0.005113705	0.067772604	Down
ROBO4	-1.035083611	0.012108116	0.119096447	Down
ROR1	-1.352092065	0.038369315	0.24374285	Down
RORA	-1.664464041	3.97729E-06	0.000312346	Down
RPL34	-1.893815612	0.003758547	0.055611297	Down
RPL6	-1.229338528	0.01759189	0.150818502	Down
RPUSD3	-1.274518311	0.000137235	0.00534005	Down
RUNX1	-1.214678891	0.001697406	0.032248218	Down
RUVBL2	-2.562493544	0.000291555	0.009195848	Down
SACS	-2.133797338	0.001370593	0.027838627	Down
SAMD12	-1.166577467	0.011254463	0.113524937	Down
SCAPER	-2.196501107	0.003051978	0.048327134	Down
SCGB2B2	-1.044006622	0.004513618	0.062515462	Down
SCMH1	-1.269046027	0.003205258	0.049918909	Down
SCML1	-1.946106867	0.001220067	0.025808342	Down
SCP2	-2.571522822	0.000879783	0.020698109	Down
SCRN1	-2.94654886	0.003456669	0.052501713	Down
SDK2	-1.031523352	0.02290768	0.177385918	Down
SEC14L3	-1.096618993	0.02725509	0.198340161	Down
SEC14L4	-1.463343238	0.024357219	0.184483617	Down
SEC16A	-1.27224008	0.046178811	0.273178864	Down
SEMA3F	-1.010011564	0.013470961	0.12734063	Down
SEPTIN2	-4.571728268	4.48598E-09	5.84005E-07	Down
SEPTIN6	-1.225886476	0.043310237	0.263506087	Down
SEPTIN7	-2.232834133	6.24277E-05	0.002997381	Down
SEPTIN8	-1.104075719	2.09633E-06	0.000182348	Down
SGCD	-1.751726951	0.000901269	0.021075786	Down
SGSH	-1.151961821	0.031710976	0.216741953	Down
SH2D1B	-1.225626138	0.000607473	0.015627935	Down
SH3BP5	-1.134496332	0.000948506	0.021825196	Down
SIAH3	-1.316107806	0.028233035	0.20272082	Down

SIGLEC1	-3.022963636	0.000112442	0.004567735	Down
SIGLEC8	-1.27815796	0.043523778	0.264200432	Down
SIRPA	-3.354336615	0.019049188	0.158539183	Down
SKA2	-1.695757914	0.01860456	0.15605706	Down
SLC12A1	-2.016336126	0.016197898	0.14294044	Down
SLC12A6	-1.147625728	0.010165881	0.107013488	Down
SLC1A2	-1.041953705	0.021664022	0.171617211	Down
SLC22A23	-1.639598052	0.047883833	0.279094548	Down
SLC25A29	-1.850106005	0.007956166	0.090829491	Down
SLC30A6	-1.15152972	0.033126474	0.22219021	Down
SLC37A2	-2.163972689	0.029320047	0.20673777	Down
SLC38A1	-1.099770786	0.000114955	0.004652729	Down
SLC38A11	-1.146210038	0.032396679	0.219686971	Down
SLC38A9	-1.693733545	0.014162437	0.13123888	Down
SLC7A8	-1.20041857	0.000980542	0.022337124	Down
SLX1B	-5.567342607	5.82556E-09	7.53342E-07	Down
SMARCA2	-1.254365153	0.015269711	0.137572795	Down
SMIM14	-2.563747402	0.000153799	0.005821102	Down
SMKR1	-2.388967786	4.80804E-06	0.000370831	Down
SMURF1	-2.255205276	0.005106274	0.067725282	Down
SNAPC5	-1.211875175	0.013182858	0.125545539	Down
SNRPD2	-1.725522831	0.001862268	0.034452409	Down
SNTG2	-1.457587221	0.006774066	0.080961145	Down
SNX19	-1.020178247	0.030200122	0.210419132	Down
SOX5	-1.18052612	0.018603209	0.15605706	Down
SOX7	-1.218992766	0.00526194	0.068895363	Down
SPG7	-1.033374419	0.02220441	0.174235456	Down
SPTSSB	-1.198653567	0.036278178	0.235826395	Down
SSR4	-4.394776323	6.4136E-06	0.000472136	Down
ST3GAL4	-1.708052446	0.03120717	0.214483093	Down
ST8SIA1	-1.374783751	0.000270277	0.00874511	Down
STOX1	-1.802202951	0.038866077	0.245451644	Down
STX2	-1.945189308	0.001116474	0.024429564	Down
STX4	-1.952269452	0.000820265	0.019669277	Down
SUGCT	-1.137054336	0.000564008	0.014804261	Down
SUGP2	-3.419795009	0.002612496	0.043517296	Down
SUN1	-1.562356187	0.012935669	0.124050199	Down
SYNCRIP	-1.441257623	0.042452414	0.260050534	Down
SYNE1	-1.415978963	0.000497596	0.013443049	Down
SYNPO	-1.125081548	0.023626227	0.180856248	Down
TACC1	-1.502972201	0.022097182	0.173675074	Down
TAF1C	-1.013197424	0.005849667	0.073501722	Down
TAL1	-1.156039934	0.039089931	0.246504207	Down
TASP1	-2.285495759	0.019565456	0.161114778	Down

TC2N	-1.068210933	0.038323939	0.243654084	Down
TCAF1	-1.496073025	0.047566838	0.278092542	Down
TCF12	-2.175530347	0.000631527	0.01607077	Down
TDRD15	-1.418579146	0.017058909	0.148086908	Down
TECR	-1.151310642	0.030431929	0.211381925	Down
TERB1	-1.772883273	9.32137E-05	0.004004681	Down
TESPA1	-1.521351981	0.008607021	0.095565631	Down
TGFBR3	-1.159420685	7.09103E-06	0.000515162	Down
TGIF1	-1.148813136	0.027786114	0.200887495	Down
THEMIS	-1.058836896	0.020651382	0.166560383	Down
THTPA	-1.978938146	0.044959749	0.268920636	Down
TIAM1	-1.059350418	0.038745229	0.244887772	Down
TIMP3	-1.149659802	0.000904417	0.02108672	Down
TK2	-3.888224258	0.00029366	0.009247186	Down
TLE4	-1.602150997	0.010198884	0.107172902	Down
TMEM102	-1.377659186	0.018485783	0.155531544	Down
TMEM156	-1.602427555	0.013817999	0.129142199	Down
TMEM168	-2.092588762	0.013509234	0.127550907	Down
TMEM176B	-2.356091051	0.003582675	0.053601961	Down
TMEM18	-1.016871792	0.002515736	0.042397043	Down
TMEM218	-1.951557467	0.001206185	0.025591711	Down
TMEM229B	-2.654697168	0.000355955	0.010565634	Down
TMTC4	-1.456368931	0.046630954	0.274537194	Down
TNFAIP8	-1.085659727	0.003859843	0.056442749	Down
TNFAIP8L1	-1.40580105	0.049629772	0.284652131	Down
TNFRSF13B	-1.058247892	0.002339484	0.040266326	Down
TNFRSF13C	-1.203302839	0.010425359	0.108718643	Down
TNFRSF18	-2.085503717	0.001712984	0.032433	Down
TNR	-1.777254616	9.9623E-07	9.5429E-05	Down
TPK1	-2.862138864	0.005699401	0.072518285	Down
TPST2	-2.153041243	0.011213306	0.113317125	Down
TRAF1	-1.504542651	0.008572576	0.095341919	Down
TRDMT1	-1.764064962	0.028549648	0.204087632	Down
TRIM65	-1.002757516	0.022004194	0.173224984	Down
TRPM3	-2.00169915	2.49121E-05	0.001433923	Down
TSBP1	-1.142644392	0.015916076	0.141439425	Down
TSPAN15	-1.231064265	6.76446E-05	0.003161775	Down
TSPAN3	-1.108075776	0.006530098	0.078871473	Down
TSPOAP1	-3.965452981	9.65058E-06	0.000655681	Down
TTC7B	-1.970819352	0.016566161	0.145244218	Down
TTN	-2.491994444	5.0019E-05	0.002500627	Down
TUBB6	-2.247170896	0.000965021	0.022100338	Down
TUBGCP5	-2.159586604	0.013681633	0.128331466	Down
TXNRD3	-1.076305889	0.021370827	0.170067946	Down

UBE2I	-2.207566963	0.007996698	0.09100378	Down
UBE3A	-2.184413794	0.001939198	0.035441704	Down
UGT2B11	-1.017424396	0.013735302	0.128617199	Down
URI1	-1.137740983	3.17846E-07	3.44437E-05	Down
URM1	-1.222654784	0.026264612	0.194046017	Down
USP10	-1.053656944	0.042340324	0.259698475	Down
VANGL2	-1.42847223	0.016964401	0.147662983	Down
VAV2	-1.449262368	0.016891603	0.147128368	Down
VIPR2	-1.637623854	0.001504123	0.029560516	Down
VOPP1	-1.105252179	0.049730944	0.28470544	Down
VPS13D	-5.170608533	1.47011E-08	1.85776E-06	Down
WNT10A	-1.216602861	0.029565937	0.2077165	Down
WNT7A	-1.074112551	0.0071858	0.084399971	Down
XRRA1	-1.47447024	0.012697596	0.122520006	Down
ZBTB25	-1.600740924	0.005715919	0.072609388	Down
ZC3H14	-2.790328247	1.39269E-06	0.00012565	Down
ZDHHC11B	-1.29316393	0.000868793	0.020501712	Down
ZFAND1	-2.220847105	0.037633447	0.241001913	Down
ZFP14	-1.25398535	0.001287208	0.02684798	Down
ZGPAT	-1.410352624	0.02986992	0.208831058	Down
ZMAT4	-2.532613706	1.13793E-05	0.00074697	Down
ZMIZ2	-1.325233544	0.008113289	0.091818853	Down
ZMYM2	-2.077134747	0.046612428	0.274488335	Down
ZMYND11	-3.590499443	0.000307596	0.009523681	Down
ZNF121	-1.233646263	0.000354472	0.010529662	Down
ZNF131	-1.753124781	0.011079341	0.112424293	Down
ZNF142	-1.370265407	0.02117889	0.169266043	Down
ZNF148	-1.667848962	0.000187502	0.006681012	Down
ZNF202	-1.611108572	0.017274959	0.149227799	Down
ZNF211	-1.529988706	0.003658787	0.054509462	Down
ZNF227	-2.263081391	0.000414457	0.011831388	Down
ZNF239	-2.511597911	0.001364221	0.027825949	Down
ZNF266	-1.19829608	0.027232197	0.198212588	Down
ZNF268	-1.698840936	0.032377438	0.219594878	Down
ZNF30	-2.544027782	0.007666438	0.088438736	Down
ZNF331	-1.470073382	0.043071468	0.262711976	Down
ZNF345	-1.256991316	0.000381751	0.011132766	Down
ZNF365	-1.006109789	0.019156307	0.159102743	Down
ZNF41	-3.289463717	0.003487092	0.0528238	Down
ZNF43	-1.051820295	0.001469035	0.029121722	Down
ZNF529	-2.305631646	0.013069066	0.12487849	Down
ZNF562	-1.152876764	0.005962539	0.074450172	Down
ZNF574	-2.017172923	0.036819043	0.237867574	Down
ZNF610	-1.335587771	0.029275498	0.206502988	Down

ZNF664	-1.153084244	0.026065741	0.193221547	Down
ZNF732	-1.181875398	0.014566624	0.133438527	Down
ZNF761	-1.260375263	0.001189998	0.025407797	Down
ZNF772	-3.741968595	3.64286E-05	0.001950646	Down
ZNF821	-1.793612491	0.004953016	0.066350912	Down
ZNF860	-1.186418918	0.000688965	0.017133587	Down
ZNF875	-1.402843845	0.003093196	0.048701524	Down
ZP1	-1.07726837	0.014072972	0.130706476	Down
ZSCAN26	-1.90777738	0.001005823	0.022819242	Down
ZZZ3	-1.669338111	0.004189713	0.059538436	Down
