# Supplementary figures and images for: Identification of Candidate mRNA and miRNA Molecules Associated with Tuberculosis Through Preliminary Analysis and Validation Using Clinical Samples
Source: Int J Mol Sci. 2026 Jun 7;27(12):5177. doi: 10.3390/ijms27125177 (PMC13299930; doi:10.3390/ijms27125177)

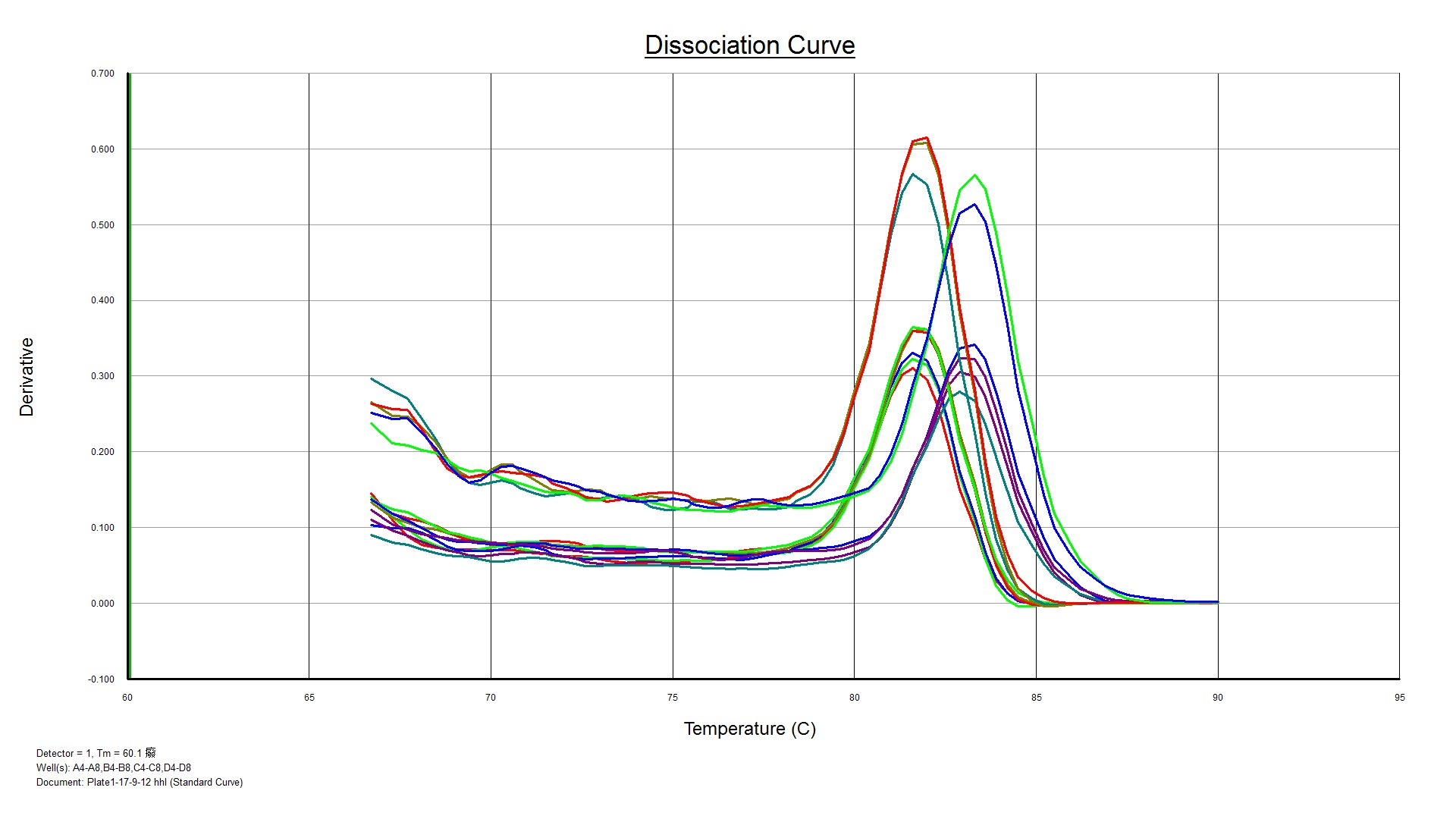

Supplement: Supplementary file 1 [file ijms-27-05177-s001.zip › Figure S1.png]
